# In silico prediction of UGT-mediated metabolism in drug-like molecules via graph neural network

**DOI:** 10.1186/s13321-022-00626-3

**Published:** 2022-07-08

**Authors:** Mengting Huang, Chaofeng Lou, Zengrui Wu, Weihua Li, Philip W. Lee, Yun Tang, Guixia Liu

**Affiliations:** Shanghai Frontiers Science Center of Optogenetic Techniques for Cell Metabolism, School of Pharmacy, East China University of Science and Technology, Shanghai, 200237 China

**Keywords:** UDP-glucuronosyltransferases, Enzyme, Drug metabolism, Glucuronidation, Phase II metabolism, Graph neural network, Weisfeiler-Lehman Networks, Consensus models, Substrate prediction, SOM prediction

## Abstract

UDP-glucuronosyltransferases (UGTs) have gained increasing attention as they play important roles in the phase II metabolism of drugs. Due to the time-consuming process and high cost of experimental approaches to identify the metabolic fate of UGT enzymes, in silico methods have been developed to predict the UGT-mediated metabolism of drug-like molecules. We developed consensus models with the combination of machine learning (ML) and graph neural network (GNN) methods to predict if a drug-like molecule is a potential UGT substrate, and then we applied the Weisfeiler-Lehman Network (WLN) model to identify the sites of metabolism (SOMs) of UGT-catalyzed substrates. For the substrate model, the accuracy of the single substrate prediction model on the test set could reach to 0.835. Compared with the single estimators, the consensus models are more stable and have better generalization ability, and the accuracy on the test set reached to 0.851. For the SOM model, the top-1 accuracy of the SOM model on the test set reached to 0.898, outperforming existing works. Thus, in this study, we proposed a computational framework, named Meta-UGT, which would provide a useful tool for the prediction and optimization of metabolic profiles and drug design.

**Graphical Abstract:**

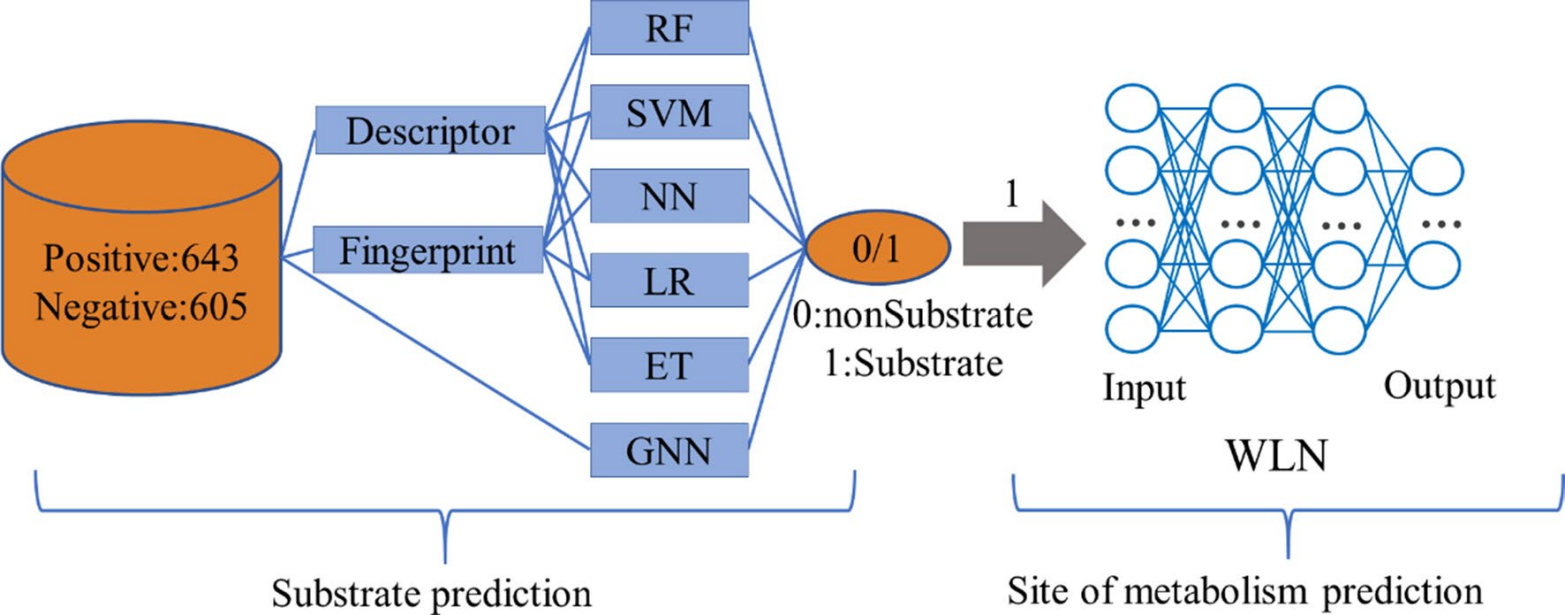

**Supplementary Information:**

The online version contains supplementary material available at 10.1186/s13321-022-00626-3.

## Introduction

**Fig. 1:**
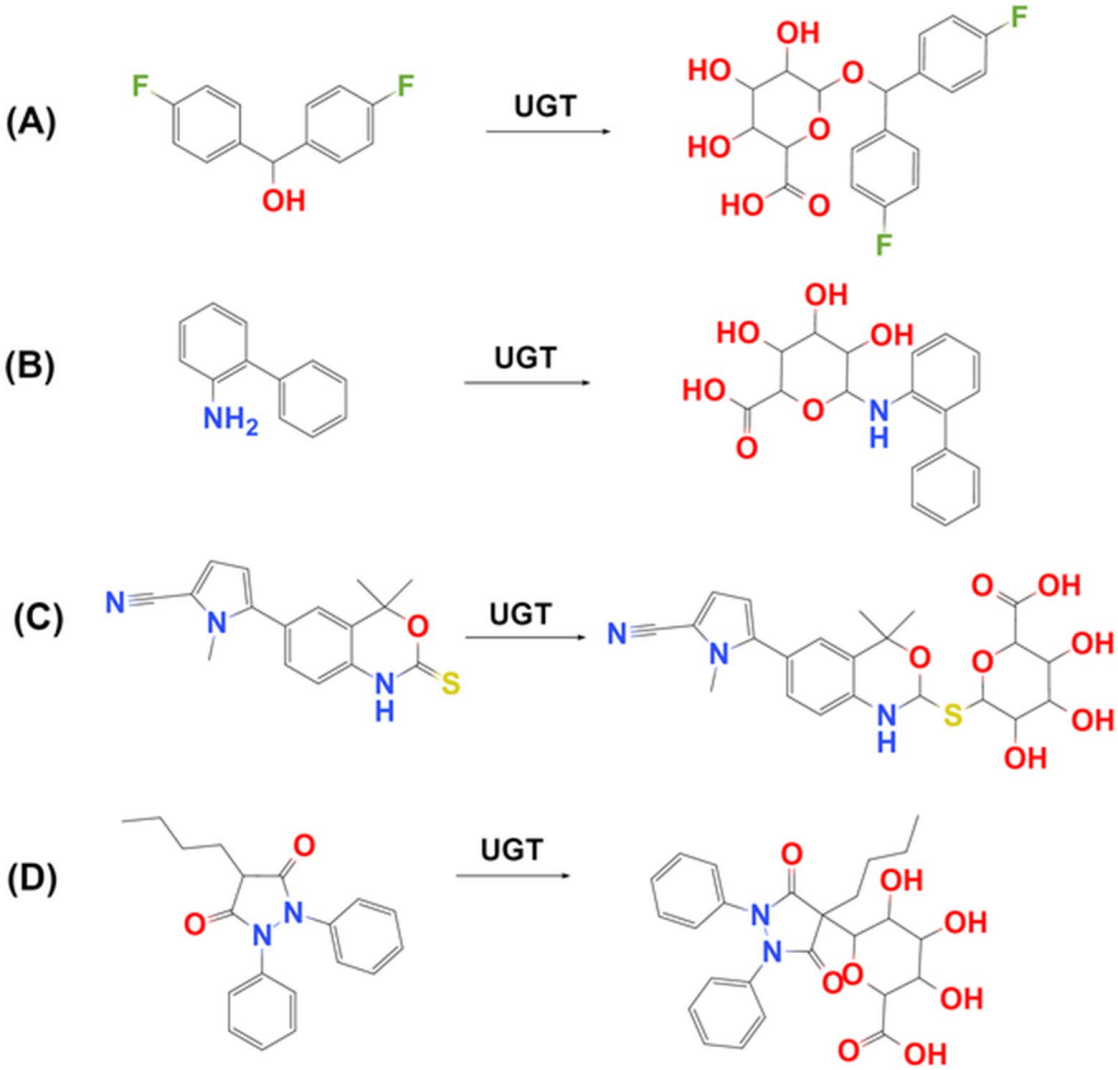
Four types of UGT catalyzed reactions. **A** O-Glucuronidation; **B** N-Glucuronidation; **C** S-Glucuronidation; **D** C-Glucuronidation

Metabolism plays a vital role in drug development, as it is one of the main clearance pathways for approximately 75% of existing drugs [1]. Metabolism can produce metabolites with different physical and chemical properties from the parent drug, which has an important impact on the safety and efficacy of the drug [2]. UDP-glucuronosyltransferases (UGTs) are the most important Phase II drug-metabolizing enzymes, including 22 members in mammals. They are classified into four subfamilies: UGT1, UGT2, UGT3, and UGT8 [3]. UGTs catalyze the conjugation of parent drugs containing a nucleophilic atom (aliphatic, aromatic hydroxyl, carboxyl, or amino groups) with glucuronic acid (Fig. 1), according to a second-order nucleophilic substitution mechanism [4].

About 15% of drugs are metabolized by UGTs [5]. Most of them are transformed into hydrophilic metabolites by UGTs for excretion. Glucuronidation can not only lead to detoxification but also cause the drug to act for a short time and lose its activity. Moreover, glucuronidation can result in the formation of more active metabolites than the parent drug, for instance, morphine produces morphine-6-glucuronide which is more potent than the parent drug through glucuronidation [6]. Taken together, determining the metabolites of UGT enzyme metabolism can help improve the metabolic properties of the drug. Experimental methods can help to explore the metabolic fate of drugs. Unfortunately, these experimental approaches have high requirements for time, equipment, manpower, and resources [7]. Therefore, computer-based methods for predicting the metabolic fate of UGT enzymes were promoted.

There were some methods developed for the prediction of sites of metabolism (SOM), and they used different features to describe the molecular environment. Peng et al. [8] calculated and selected molecular descriptors of atom reactivity, bonding strength, and physical–chemical properties via a genetic algorithm-based feature selection approach to predict sites of glucuronidation. In our previous work, Cai et al. used atom environment fingerprints which represent the potential site in a molecule to predict the site of metabolism (SOM) for UGT-catalyzed reactions [9]. The above two methods need to find out all potential sites of metabolism (SOMs) and then mark whether they are SOMs. Rudik et al. developed and applied a method—SOMP [10], which was based on LMNA descriptors and the PASS algorithm to predict sites of metabolism. This method needs to calculate descriptors by the PASS algorithm. Dang et al. computed a vector of topological, molecule, quantum chemical, and statistical descriptors to represent each atom in a molecule [11]. Their methods need to calculate technically complex quantum-chemical-derived descriptors. All of them only built a model to predict the site of metabolism but did not consider if the molecule was the substrate of UGT enzymes. Most of these methods need to calculate complex descriptors by other tools to predict the SOM for UGT-catalyzed reactions. Meanwhile, we found that most of these models were built on the substrates data, and some nonsubstrates fell outside the applicability domain of SOM model. Thus, the substrate/nonsubstrate prediction model can improve the performance on nonsubstrates. If a molecule is not the substrate of UGTs, it is not necessary to predict SOM. Therefore, it is necessary to build a model which can predict if a molecule is the substrate of the UGT enzyme and then predict the SOM of the substrate.

Molecular representation is a vital point in metabolic prediction. Molecules were often presented as physicochemical descriptors or fingerprints. Recently, graphs were used to describe molecular structures; atoms were represented as nodes, and bonds were represented as edges [12, 13]. In fact, graph theory has been applied to solve many chemical questions including the representation of chemical reactions. Duvenaud et al. used graphs to represent molecules in chemistry by developing neural fingerprints [14]. Convolution operation rather than hashing function was used to assemble features of neighboring nodes. Kearnes et al. described a molecular graph convolutions architecture for learning from small molecules [15]. They proposed that the model will take greater advantage of the information in the graph convolution method than fingerprint-based methods which focus on particular aspects of the molecular structure. They expected the graph convolution method could have better performance than fingerprint-based methods through optimizations. Coley et al. developed a novel approach based on Weisfeiler-Lehman Network (WLN) to predict chemical reactivity [16]. The Weisfeiler-Lehman kernel [17] could capture graph transformation of reactions. Thus, given reactants and reagents of the organic reactions, the WLN model could predict the products by learning to identify possible reactive sites.

In this work, there are two objectives: (1) to accurately predict if a molecule is the substrate of UGT enzymes, and (2) to predict where the SOMs are located, i.e., to identify the specific site within the substrate that is metabolized by UGTs. We first applied the combination of fingerprint-based and physicochemical descriptors-based ML methods and the GNN methods to predict if the molecule is a substrate of UGT. The WLN method was applied to predict potential SOM in the substrate. The model scored the reactivity between atom pairs and predicted the site of metabolism. The GNN methods need not calculate complex quantum chemical descriptors by other descriptors programs. It was the first time to use the graph method in the prediction of SOM. In this study, we proposed a computational framework, named Meta-UGT, to predict the substrate of UGT enzymes and their SOMs.

## Materials and methods

### Data collection and preparation

All the substrate/nonsubstrate data of UGT enzymes were collected from publications (listed at https://github.com/mengtinghuang/Meta-UGT). If a compound is determined to be metabolized by UGT enzymes as it has the glucuronidation metabolites, we labeled it as “substrate”. All chemical structures were saved as Simplified Molecular Input Line System (SMILES) String. The dataset was randomly split into training and test sets (8:2).

All the UGT-catalyzed reactions were collected from literature and most of the data were retrieved from Lee’s Handbook of Metabolic Pathways of Xenobiotics by Cai et al. [9]. All four types of UGT catalyzed reactions as shown in Fig. 1 were collected were aimed to build a global SOM model However, we found that there are very few data on S-Glucuronidation and C-Glucuronidation. Therefore, we only considered O-Glucuronidation and N-Glucuronidation here. All SOMs were classified into four types of substructure groups, These groups were aliphatic hydroxyls (AlOH), aromatic hydroxyls (ArOH), carboxylic acids (COOH), and nitrogen containing sites (Nitrogen). Each reaction datum was restored as reaction SMILES including the information of reactants and products as shown in Table 1. The dataset was randomly split into training, validation, and test sets (8:1:1). Metabolic reactions with the same reactant, but different metabolites were split in the same set.

**Table 1:** Data preparation steps for the SOM model

Step	Example: reactants >> metabolites
Reaction	
Original SMIRKS	O=C(C1=CC(Br)=CC(Br)=C1N)O.O=C(C2OCC(C(C2O)O)O)O >> BrC3=CC(Br)=C(C(C(OC4OC(C(O)C(O)C4O)C(O)=O)=O)=C3)N
Atom-Mapping SMIRKS	[O:1]=[C:2]([OH:3])[C:4]1=[CH:5][C:6]([Br:7])=[CH:8][C:9]([Br:10])=[C:11]1[NH2:12].[O:13]=[C:14]([OH:15])[CH:16]1[O:17][CH*:18][CH:19]([OH:20])[CH:21]([OH:22])[CH:23]1[OH:24] >> [Br:7][C:6]1=[CH:8][C:9]([Br:10])=[C:11]([NH2:12])[C:4]([C:2]([O:3][CH:18]2[O:17][CH:16]([C:14]([OH:15])=[O:13])[CH:23]([CH:21]([CH:19]2[OH:20])[OH:22])[OH:24])=[O:1])=[CH:5]1

For the SOM prediction model, we should preprocess the data before building the WLN model. All the reactions were saved as SMIRKS, and then we added a map for the SMIRKS by RDT software [18]. The data preprocessing steps were shown in Table 1. The SMIRKS include reactants and metabolites, and they are split by ‘>>’. If the reaction has two or more reactants, they will split by ‘.’. In order to add the map successfully for the SMIRKS, the reaction equation needs to be balanced. The mechanism of glucuronidation was shown in Additional file 1: Fig. S1. Under the action of UDP-glucuronosyltransferases, the nucleophilic substrate will attack the glucuronic acid, and release one molecule of Glucuronide conjugate and one molecule of UDP. In order to reduce the complexity of prediction, we do not take the effect of uridine diphosphate into consideration as each glucuronidation reaction will release one.

### Calculation of molecular fingerprints and physicochemical descriptors and definition of graph features

Five types of fingerprints that have been widely used in QSAR modeling were used to characterize compounds, and they were generated by the open-source toolkit RDKit (version 2018.09.3.0) [19] (http://www.rdkit.org/): (I) AtomPairs (512, 1024, 2048 bits); (II) MACCS (166 bits); (III) Morgan (512, 1024, 2048 bits); (IV) Topological Torsions (TopoTorsion, 512, 1024, 2048 bits); and (V) RDKit (512, 1024, 2048 bits). These fingerprints were used to build traditional ML models. For AtomPairs, Morgan, TopoTorsion, and RDKit fingerprints, we tried three different bit lengths (512, 1024, 2048 bits) and then chose the best one for molecular representation. The radius of Morgan fingerprints was the default (radius = 2). We also tried to use the physical and chemical descriptors to build traditional ML models. 729 physical and chemical descriptors were calculated by the PaDEL-Descriptor software [20].

We used different GNN methods to construct the substrate prediction models and SOM model. The molecular graph was considered as the input of models. We need to define the molecular features in advance as the atoms are represented as nodes and the bonds are represented as the edges. The definition of the atom and bond features was shown in the supporting information (Additional file 1: Tables S1–S6). We feed the model with SMILES, and then the model will transform the structure into one-hot encoding by the definition. In this way, any descriptors did not need to be calculated by other tools.

### Model construction

#### Model for substrate/nonsubstrate classification

Five traditional ML methods and five GNN methods were used to construct substrate prediction models. The traditional ML methods include random forest (RF), support vector machine (SVM), logistic regression (LR), neural network (NN), and extremely randomized trees (ET), while the GNN methods are graph convolutional networks (GCN), graph attention networks (GAT), weave (Weave), message passing neural network (MPNN), and attentive FP networks (Attentive FP).

##### Traditional ML methods

RF refers to a special bagging method that fits several decision tree classifiers on various sub-samples of the dataset, and it uses averaging to improve the predictive accuracy and avoid over-fitting [21]. SVM is a class of generalized linear classifiers that perform binary classification of data in a supervised learning method. Its decision boundary is the maximum margin hyperplane that is solved for the learning sample [22]. LR is a linear model for classification instead of regression. Logistic regression is also known in the literature as logit regression, maximum-entropy classification (MaxEnt), or the log-linear classifier [23]. Inspired by biological nerves, people invented artificial NN based on Multi-Layer Perceptron (MLP), which is a supervised learning algorithm that learns optimal parameters by training on a dataset. Geurts et al. proposed ET for supervised classification problems [24]. It constructs completely randomized trees whose structure is not affected by the output value of the learning sample.

##### Graph Convolutional Networks (GCN) and Graph Attention Networks (GAT)

For GNN methods, the forward pass has two stages: one is "Message Passing" and the other is "Readout". These GNN methods can be used for substrate classification based on graphs rather than fingerprints. Atom represents a node. After updating node representations by adjacent atoms, this method performs a weighted sum with learnable weights and max-pooling layer and concatenates the output of the two operations, which is then fed into an MLP for final prediction. For the GAT, the attention mechanism is employed to update the node representations on the basis of the GCN.

##### Weave and Message Passing Neural Network (MPNN)

A node represents an atom, and an edge represents a bond. Not only the impact of the node but also the edge is considered by the two methods to generate molecule-level features. The weave used a method similar to histograms to replace the operations of sum, mean, and max to model the distribution of data in each dimension [15]. Message Passing Neural Networks (MPNN) reformulated some existing models into a single common framework and explore additional novel variations within this framework.

##### Attentive FP networks [25]

Atom features and bond features were both used to build the Attentive FP model. Attentive FP can effectively capture the non-local features of the graph and the interaction of distant nodes while maintaining the inherent structure of the molecule. This is because Attentive FP firstly adds the attention mechanism at the atomic level to learn the local characteristics of the molecule, and then adds the attention mechanism at the entire molecular level to learn the global characteristics of the molecule.

##### Consensus models

According to the performance of tenfold cross-validation, we would select the top-10 models to build consensus models. We combined n models from the top-10 models to construct consensus models (n = 2, 3, 4, 5, 6, 7, 8, 9, 10). Finally, we totally built 1023 consensus models (${C}_{10}^{2}+{C}_{10}^{3}+{C}_{10}^{4}+{C}_{10}^{5}+{C}_{10}^{6}+{C}_{10}^{7}+{C}_{10}^{8}+{C}_{10}^{9}+{C}_{10}^{10}=1023$). The consensus models were built by the soft voting strategy. The final output was determined by the average value of probability from all single models. If the average probability ≥ 0.5, the samples were predicted as the substrate of UGT.

#### Model for SOM prediction

In the prediction model for SOM, we applied the framework of the organic reaction prediction method developed by Coley for metabolic prediction [16].

##### Weisfeiler-Lehman Networks (WLN)

**Fig. 2:**
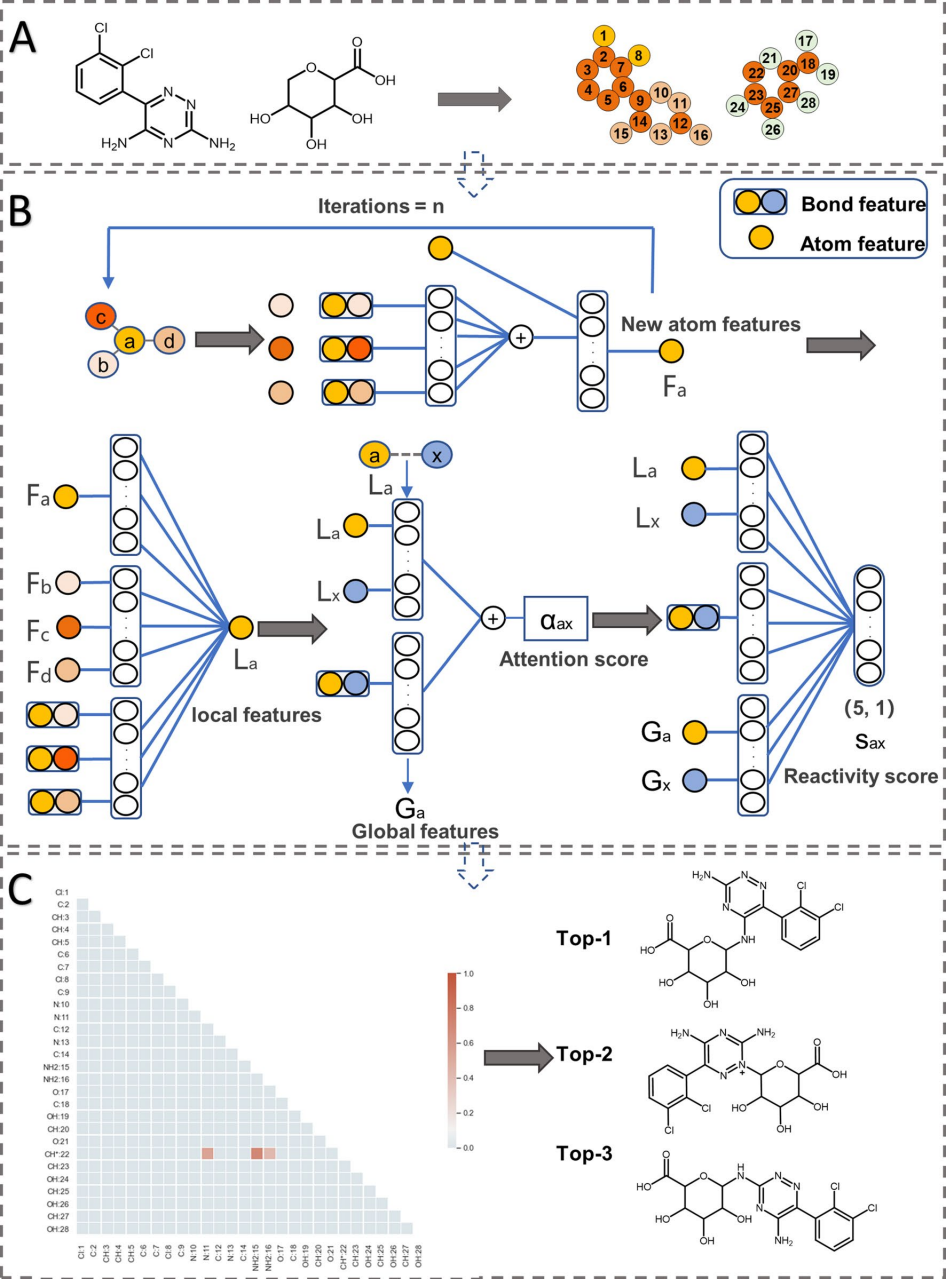
The workflow of the WLN approach to predict SOMs of UGT enzyme. **A** Graph representation of molecules; **B** WLN model for predicting the possible site of metabolism by calculating reactivity scores; **C** the visualization of the reactivity scores of possible sites

The UGT-catalyzed reaction is considered as a set of graph edits where the edge between the node of the drug and the node of the glucuronic acid is changed. The key to the success of the prediction is to learn a representation of the molecules that captures properties relevant to the atom environment. As shown in Fig. 2A, we feed the WLN model with atom-mapping reaction SMIRKS. It was worth mentioning that the nonsubstrates could not generate SMIRKS, so the WLN model was based on the data of substrates. The model will extract the information of the atoms and bonds which were described as nodes and edges. Node descriptors including atom type, atom degree, atom explicit valence, atom implicit valence, and aromaticity and edge descriptors including bond type, whether the bond is conjugated, and whether the bond is in-ring were considered. The definitions of all the atom and bond features in this work are available in the supporting information (Additional file 1: Table S5 and S6). The process of iteratively updating atom-level representations is shown in Fig. 2B. Firstly, the information of the center atom (“a”), adjacent atoms (“b”, “c”, “d”), and bonds (“ab”, “ac”, “ad”) is merged by a parameterized neural network to generate new atom features for the center atom “a”, and this process may be iterated several times. Next, the new atomic features and their neighbors’ updated features will be used to calculate the local features of the atom. In order to consider the effect of other molecules, a global attention mechanism is added to this model to produce a context vector for each atom [26]. Finally, a combination of local features and global features is used to predict the possibility of the site of metabolism of UGT enzymes. As shown in Fig. 2C, the WLN model will learn the local features and global features to calculate the reactivity scores of possible SOMs of UGT enzymes. The reactivity score is higher, the site is more likely to happen glucuronidation. When we know the site of metabolism, we can know the metabolite of the UGT enzymes as the mechanism of glucuronidation shown in Additional file 1: Fig. S1.

### Tuning parameters and early stopping for GNN models

The parameters of GNN models can be divided into two categories: model parameters and hyperparameters. Model parameters are updated by the gradient descent algorithm during training. GNN methods mentioned were trained with the PyTorch framework and the model parameters were updated by Adam optimizer for gradient descent optimization. Hyperparameters are generally fixed values or changed according to preset rules during training, such as batch size, learning rate, weight decay, etc. There are some tuning methods such as grid search, random search, and Bayesian optimizations, which are commonly used algorithms. We used Bayesian optimization to obtain the best hyperparameters for the GNN methods [27]. BCEWithLogitsLoss which measures cross-entropy as loss functions were employed for the classification tasks.

In order to avoid overfitting and save training time and training resources, early stopping [28] was used in the substrate prediction model based on GNN methods. When using Bayesian optimization to search for hyperparameters, a training process is required to obtain the best performance. In this training process, we set a maximum epoch of 500, and if the performance metric had not improved in 10 epochs on the training set and in 15 epochs on the validation set, the training process was terminated early. All of the GNN models were trained until the performance improvement had converged.

### Evaluation of model performance

In our substrate classification task, the classification accuracy (ACC), sensitivity (SE), specificity (SP), Matthews correlation coefficient (MCC), and area under the curve (AUC) were employed to evaluate the performance of a classifier with tenfold cross-validation. These metrics are calculated based on true positive (TP), false positive (FP), true negative (TN), and false negative (FN). The first four metrics were calculated using the following equations:

1 $$\mathrm{ACC}= \frac{TP+TN}{TP+TN+FP+FN}$$

2 $$\mathrm{SE}=\frac{TP}{TP+FN}$$

3 $$\mathrm{SP}=\frac{TN}{TN+FP}$$

4 $$\mathrm{MCC}=\frac{TP*TN-FP*FN}{\sqrt{(TP+FP)(TP+FN)(TN+FP)(TN+FN)}}$$

For the SOM model, we calculated the AUC, MCC and “Top-k” metrics to evaluate. Let ${\widehat{f}}_{i,j}$ be the j-th predictive result for the i-th molecule, y_i_ be the actual value, and n_samples_ be the number of substrates, the top-k accuracy can be written as:

5 $$\mathrm{Top}-\mathrm{k accuracy}\left(\mathrm{y}, \widehat{f}\right)=\frac{1}{{n}_{samples}}\sum_{i=0}^{{n}_{samples}-1}\sum_{j=1}^{k}1({\widehat{f}}_{i,j}={y}_{i})$$

A substrate was considered to be correctly predicted if any of its experimentally observed sites of metabolism were predicted in the top k rank positions out of all potential sites in the substrate.

### Comparison with existing models

Several known models can predict the SOMs of the molecule by UGTs. We compared our model with SOMP [10] (http://www.way2drug.com/SOMP), FAME3 [29] (https://nerdd.univie.ac.at/fame3/) and XenoSite [11] (https://swami.wustl.edu/xenosite) on the same test set. We input the test set to the above web servers and then got the predictive results. We also compared our present work with our previous work completed by Cai et al. [9]. We compared our model with Cai’s global model whose method combination included the AdaBoost classifier without the resampling operation and 255 components for the PCA method.

## Results

### Data set analysis

A total of 1248 data were collected, including 643 substrate data and 605 nonsubstrate data. The substrate was labeled as a positive compound labeled as “1”, and the nonsubstrate was considered as a negative compound labeled as “0”. In order to verify the reliability of the data, y-Randomization [30] was applied to randomize the label (“0” or “1”) of the data and to see if they can obtain models. We can find the randomized model is worse than the original model as shown in Additional file 1: Fig. S2, which indicated our data is reliable. Altogether 652 UGT-catalyzed reactions were collected from literature and all the reactions were saved as SMIRKS. 536 SMIRKS were split as a training set to train the WLN model, 57 SMIRKS were split as a validation set to select the best model and 59 SMIRKS were split as a test set to evaluate the best model.

**Fig. 3:**
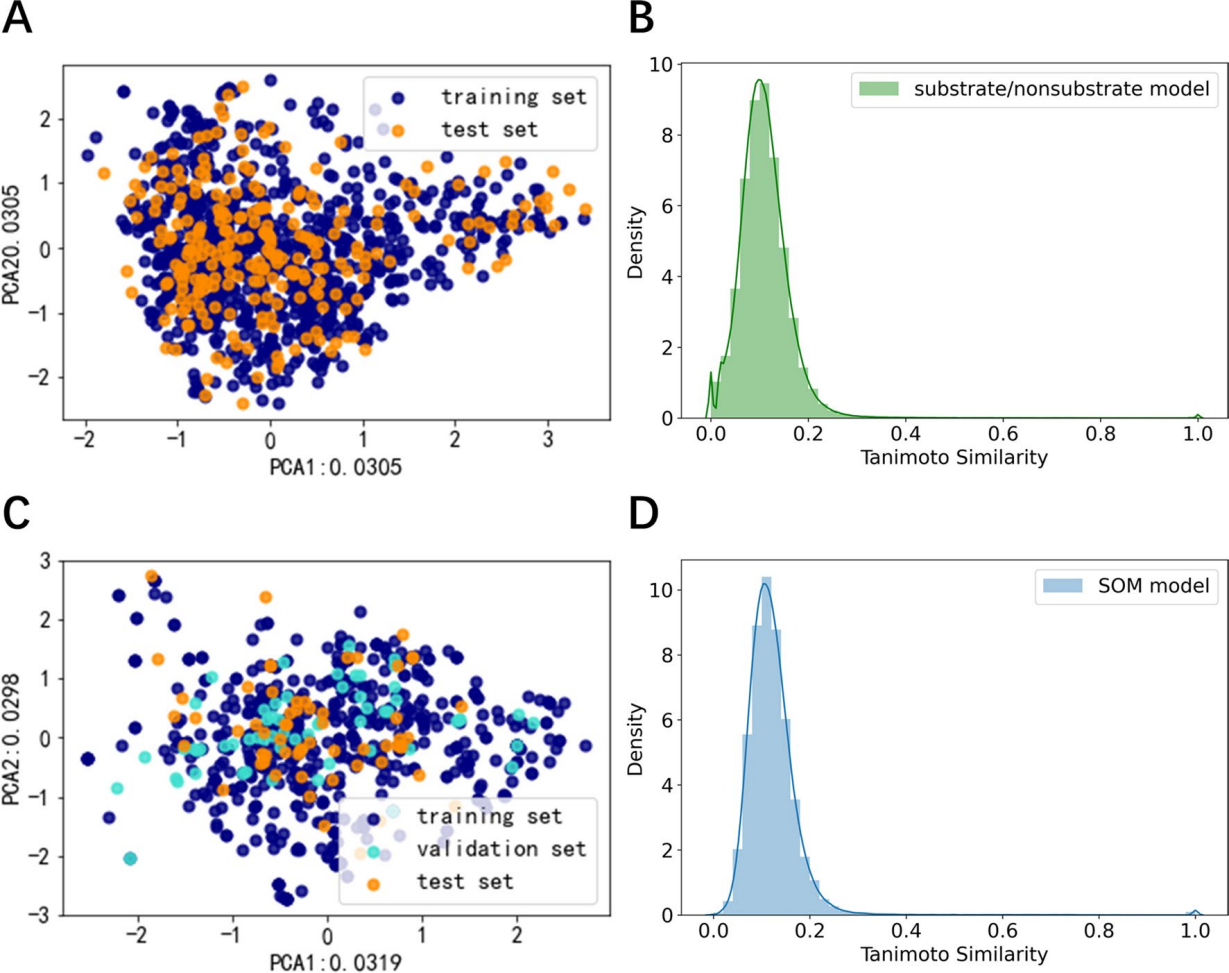
The chemical space of the data. **A** PCA analysis of substrate and nonsubstrate data based on Morgan fingerprints; **B** Tanimoto similarity of the substrate and nonsubstrate data based on Morgan fingerprints; **C** PCA analysis of the data for SOM model based on Morgan fingerprints;** D** Tanimoto similarity of the data for SOM model based on Morgan fingerprints

As shown in Fig. 3, we used two-dimensional principal component analysis (PCA) based on the Morgan fingerprint to explore the chemical space distribution of the different datasets. We calculated the Tanimoto coefficient based on the Morgan fingerprint[31] to calculate the similarity of our collected data to further explore the chemical diversity. As shown in Fig. 3A and C, we performed PCA analysis of substrate/nonsubstrate data and SOM data, respectively. In general, the distribution of all test set and validation set was roughly within the scope of the chemical space of the training set, which indicated that our model could predict the structure of the test set. We also calculated the Tanimoto similarity among training set and test set for the substrate/nonsubstrate model and SOM model as shown in Additional file 1: Fig. S3. As shown in Additional file 1: Fig. S3A and B, most of the data in the test set had similar data in the training set. Moreover, the average value overall maximum Tanimoto similarities between the data in test set and training set was 0.561 for the substrate model and 0.541 for the SOM model, which further supported the conclusion that our model could predict the structures of the test set. Histograms of frequency distribution of similarity for chemicals of the substrate/nonsubstrate model and SOM model were shown in Fig. 3B and D. As shown in Fig. 3B and D, the Tanimoto similarity between compounds was mostly distributed between 0 and 0.4. The average Tanimoto similarity of the substrate and nonsubstrate data set was 0.123 and the average Tanimoto similarity of the substance with known metabolic sites by UGT enzymes was 0.110, which indicated that compounds used in our research were obviously structurally diverse.

### Performance of the substrate prediction model

#### Performance of tenfold cross-validation

**Fig. 4:**
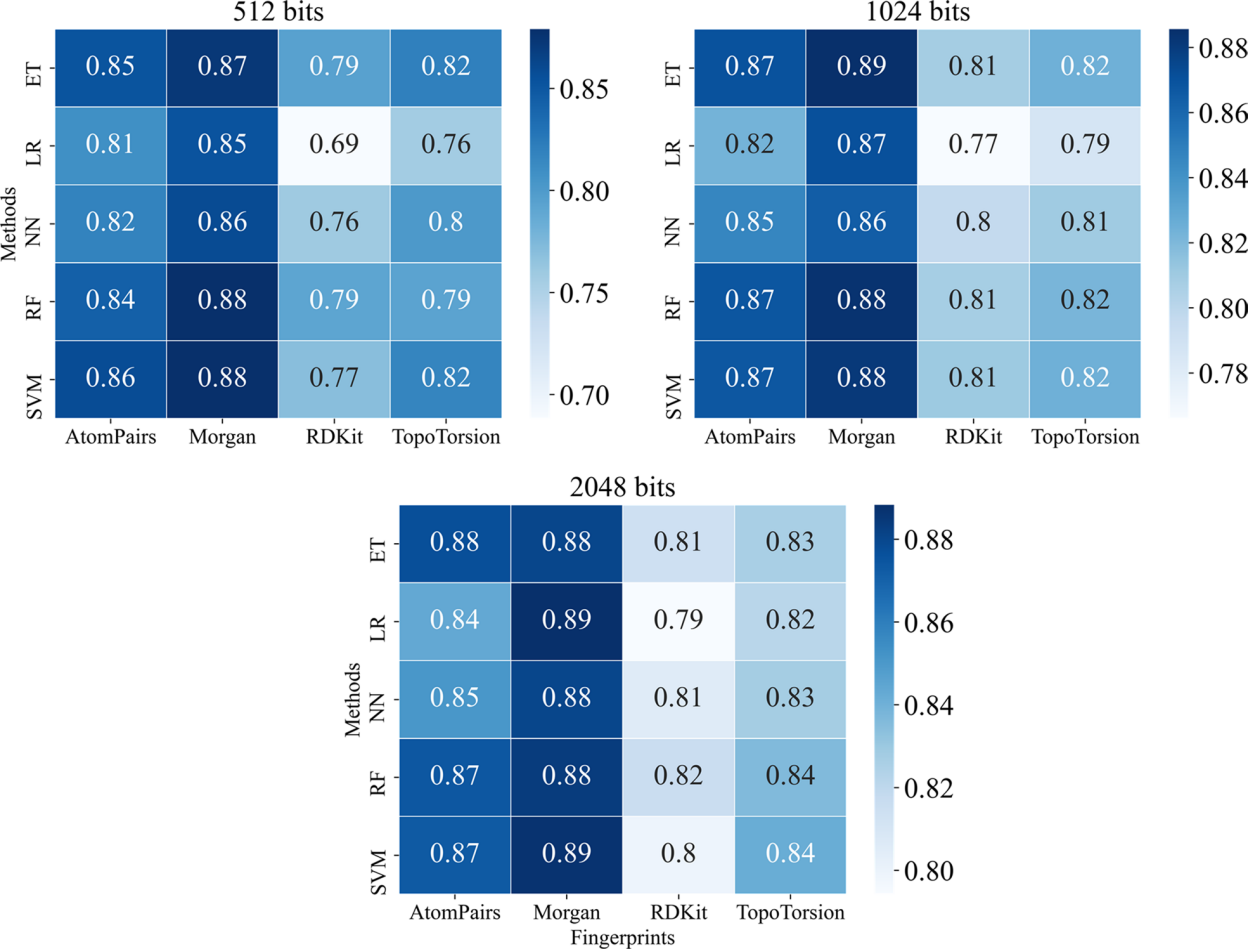
The AUC value of five machine learning methods (SVM, RF, NN, LR, ET) in different bits (512, 1024, and 2048 bits) of four types of molecular fingerprints (AtomPairs, Morgan, RDKit, and TopoTorsion) in tenfold cross-validation for the substrate prediction model

**Fig. 5:**
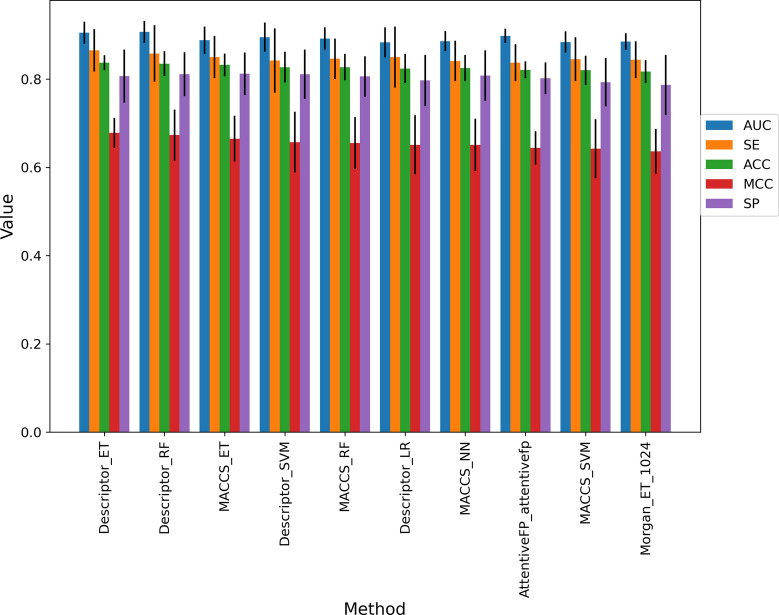
The performance of the top-10 models selected from tenfold cross-validation for the substrate prediction model. (Methods name includes the type of features and type of machine learning methods.)

Five traditional ML methods combined with five fingerprints and physicochemical descriptors were used to predict if the drug is the substrate of UGT enzymes. We performed a tenfold cross-validation and grid search to find the best parameters and the optimal traditional ML model. Some important parameters for the traditional ML model were optimized. Grid search was applied to find the best parameters, while not every parameter was tuned. We tuned the parameters based on prior published work [32]. The GNN model search for hyperparameters through Bayesian optimization on training data. The list of parameters that were tuned for the traditional ML models was showed in Additional file 1: Table S7. The tuned parameters of each traditional ML model were displayed in Additional file 1: Table S8, and the tuned parameters of each GNN model were listed in Table S9. The performance of the training set was listed in Table S10. The performance of the tenfold cross-validation was shown in Figs. 4 and 5. As shown in performance of training set and tenfold cross-validation, we found that the model was not overfitting and had good robustness.

The MACCS fingerprint was fixed at 166 bits, but the other four fingerprints had different sizes. The results of AUC in 512, 1024, and 2048 bits were shown in Fig. 4. Overall, the fingerprints of 512 bits are not as good as fingerprints of 1024 and 2048 bits except for the Morgan fingerprint. The AtomPairs and Morgan fingerprints had better performance than RDKit and Topological Torsions fingerprints. Morgan fingerprint performed best among the four molecular fingerprints. It turned out that random forest and extreme trees models performed well, which are both tree-based ensemble models.

According to the performance of tenfold cross-validation, we selected the top-10 models based on the value of MCC, which were shown in Fig. 5. The performance of other models was shown in Additional file 1: Table S11, and the MCC values for all models were distributed between 0.320 and 0.678, which indicated a large variance in model performance. As shown in Fig. 5, we found that the models based on MACCS fingerprint and physicochemical descriptors had better performance. All the top-10 models have good predictive performance, and the average AUC values were above 0.88. The top-3 models were Descriptor_ET (MCC = 0.678, AUC = 0.905, ACC = 0.837, SE = 0.865, SP = 0.807), Descriptor_RF (MCC = 0.673, AUC = 0.907, ACC = 0.835, SE = 0.858, SP = 0.811), and MACCS_ET (MCC = 0.665, AUC = 0.888, ACC = 0.832, SE = 0.850, SP = 0.812).

#### Performance of test set

In order to further explore the performance of the models, we trained traditional ML models and GNN models to obtain the optimal model through the performance of tenfold cross-validation and then predicted the results on the test dataset to evaluate these models. The performance of the top-10 models in the test dataset was listed in Table 2 and the results of the remaining models were listed in Additional file 1: Table S12, and as the results shown, all the models had good generalization ability on the test set. The performance of top_3 models based on tenfold cross-validation in the test set were Descriptor_ET (MCC = 0.628, AUC = 0.883, ACC = 0.815, SE = 0.891, SP = 0.736), Descriptor_RF (MCC = 0.616, AUC = 0.889, ACC = 0.811, SE = 0.859, SP = 0.760), and MACCS_ET (MCC = 0.616, AUC = 0.859, ACC = 0.815, SE = 0.859, SP = 0.769), respectively. As shown in Table 2, the top-10 models had good generalization ability in the test set, and the AUC values of the top-10 models in the test set were also above 0.85.

**Table 2:** The performance of the top-10 models in the test dataset for the substrate prediction model

Model	ACC	SP	SE	AUC	MCC
Descriptor_ET	0.815	0.736	0.891	0.883	0.628
Descriptor_RF	0.811	0.760	0.859	0.889	0.616
MACCS_ET	0.815	0.769	0.859	0.859	0.616
Descriptor_SVM	0.811	0.744	0.875	0.882	0.633
MACCS_RF	0.807	0.727	0.883	0.864	0.619
Descriptor_LR	0.795	0.744	0.844	0.877	0.591
MACCS_NN	0.807	0.760	0.852	0.870	0.615
AttentiveFP_attentivefp	0.823	0.785	0.859	0.877	0.647
MACCS_SVM	0.803	0.744	0.859	0.850	0.608
Morgan_ET_1024	0.807	0.727	0.883	0.878	0.619

#### Performance of consensus models

In order to improve the stability and robustness, we built consensus models. According to the performance of tenfold cross-validation, we selected the top-10 models as shown in Fig. 5. We combined n models from the top-10 models to construct consensus models and totally built 1023 consensus models. According to the value of MCC in the test set, we selected the top-3 consensus models which were listed in Table 3. The top-1 consensus model was the model combined Descriptor_SVM, Descriptor_ET, MACCS_RF, AttentiveFP_attentivefp and Morgan_ET_1024. Compared with the single model as shown in Table 2, the top-1 consensus model had a better performance (MCC = 0.682, AUC = 0.898).

**Table 3:** The results of different consensus models in the test dataset for the substrate prediction model

No	Consensus models	AUC	MCC
1	Descriptor_SVM	0.898	0.682
	Descriptor_ET		
	MACCS_RF		
	AttentiveFP_attentivefp		
	Morgan_ET_1024		
2	MACCS_ET	0.886	0.682
	Descriptor_SVM		
	MACCS_RF		
	AttentiveFP_attentivefp		
3	Descriptor_ET	0.897	0.674
	Descriptor_RF		
	MACCS_ET		
	MACCS_RF		
	AttentiveFP_attentivefp		
	Morgan_ET_1024		

In order to further explore the justifiability of the consensus model, we tried to build consensus model only with traditional ML model or GNNs. The best consensus model built only with traditional ML models was combined Descriptor_SVM, MACCS_RF, MACCS_SVM, and Morgan_ET_1024. The best consensus model built only with traditional ML models (AUC = 0.886, MCC = 0.674) performed better than the best single model (AUC = 0.883, MCC = 0.628), while performed worse than the top-1 consensus model (AUC = 0.898, MCC = 0.682), and the model spent 0.67 s in predicting the test set on 64 CPUs. The best consensus model built only with GNNs models was combined with MPNN_canonical and AttentiveFP_attentivefp (AUC = 0.877, MCC = 0.633), which even performed worse than the best single GNN model (AUC = 0.877, MCC = 0.647), and the model spent 17.37 s in predicting the test set on 64 CPUs.

Interestingly, consensus model which combined traditional ML models and GNNs (AUC = 0.898, MCC = 0.682) can improve the performance than the best consensus model built only by traditional ML models (AUC = 0.886, MCC = 0.674) or GNNs. Finally, we chose the top-1 consensus model (shown in Table 2) as the substrate prediction model. The top-10 models spent 0.06–5.26 s in predicting the test set on 64 CPUs, while the top-1 consensus model spent 9.54 s.

### Performance of the SOM prediction model

We applied the WLN model which is a GNN method developed by Coley to predict the SOMs of the UGT enzymes.

#### Various iterations and data size

**Fig. 6:**
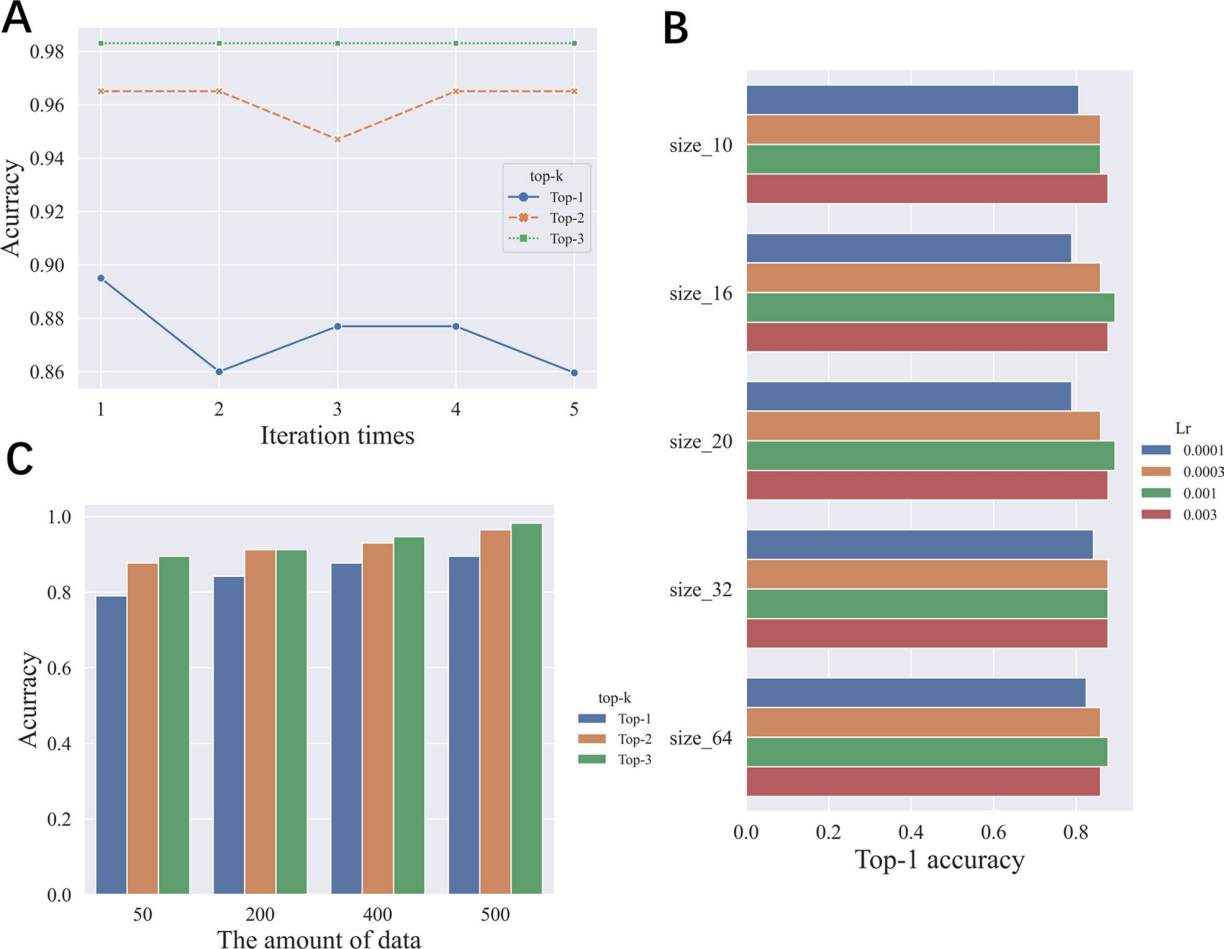
**A** The performance of top-k accuracy in different iterations of message passing (n_layers = 1, 2, 3, 4, 5); **B** different batch size (10, 16, 20, 32, 64) and learning rate (0.0001, 0.0003, 0.001, 0.003) were employed to train the SOM prediciton model; **C** the top-1, top-2 and top-3 accuracy was used to assess and compare all of these models

For the SOM prediction model, we tuned the parameters including the batch size, learning rate, and iteration layers by gird searching. As shown in Fig. 6, we trained our model in different iterations, learning rates, and batch sizes to find optimal parameters and the optimal model. As shown in Fig. 6A, we trained our model in different iterations to obtain new atom features. We found that the model would get better results when iteration times were one. What we can see in Fig. 6B is that the learning rate has a great impact on the model performance. We set the learning rate to 0.0001, 0.0003, 0.001, and 0.003, which were the common values of the learning rate. Meanwhile, because our data size is not big, we did not consider the big batch size and we set the batch size to 10, 16, 20, 32, and 64. From the perspective of the learning rate, when the learning rate is set to 0.0001, the model performs the worst. We found that the model would get a better result when the learning rate was set to 0.001 and the batch size was set to 20. As shown in Fig. 6C, we trained our model in different data sizes. When the training set data was only as small as 50, the result of top-1 accuracy can still reach more than 0.75, demonstrating the powerful predictive performance of the WLN method. As the amount of data in the training set increased, the top-1, top-2, and top-3 accuracy of the model gradually increased. When the amount of data exceeded 200, the value of the top-1 accuracy could reach above 0.8. The final parameters setting of WLN was listed in Additional file 1: Table S13.

#### Modeling results and comparison with existing models

In the present work, we applied the WLN method to predict the SOMs of UGT enzymes. We used the validation dataset to early stop. To compare our model with others, we predicted the SOM of our molecules on the test set by SOMP, FAME3, XenoSite, and Cai’s work. The results of our model with the previous model were shown in Table 4.

**Table 4:** The top-1, top-2, top-3 accuracy, AUC and MCC of the WLN model compared with others in the test set

Accuracy	Top-1	Top-2	Top-3	AUC	MCC
Our work	0.898	1.000	1.000	0.995	0.844
Cai’s work	0.719	0.797	0.814	0.874	0.558
SOMP	0.814	0.950	0.967	–	–
XenoSite	0.864	1.000	1.000	0.996	0.842
FAME3	0.814	0.898	0.932	0.977	0.645

As we can see in Table 4, the accuracy of top-1, top-2, and top-3 of our global model was better than the others. The SOMP, XenoSite, and Cai’s work specifically predicted the SOM for UGT-catalyzed reactions, while FAME3 was not specific for glucuronidation, and this model can make more general predictions on phase II metabolism. This may be one reason why FAME3 was inferior to our model. The most important metric of the results is top-1 accuracy as most of the substrates have only one SOM for UGT-catalyzed reactions, and the top-1 accuracy of our model reached 0.898. We can accurately predict all possible sites of metabolism of UGT enzymes in the top-2 rank positions, so our top-2 accuracy can reach 1.00 on the test set, showing the powerful prediction performance of our model.

The statistical information for the three data sets of four types of UGT-catalyzed reactions (AlOH, ArOH, COOH, Nitrogen) was listed in Table S14. We calculated the metrics in different types of UGT-catalyzed reactions. We found that the model performance of AlOH (Top-1 Acc = 1.000), ArOH (Top-1 Acc = 0.900), COOH (Top-1 Acc = 0.882), and Nitrogen (Top-1 Acc = 0.750) was different. As the results shown, it performed better on the types of hydroxyls group than others.

**Fig. 7:**
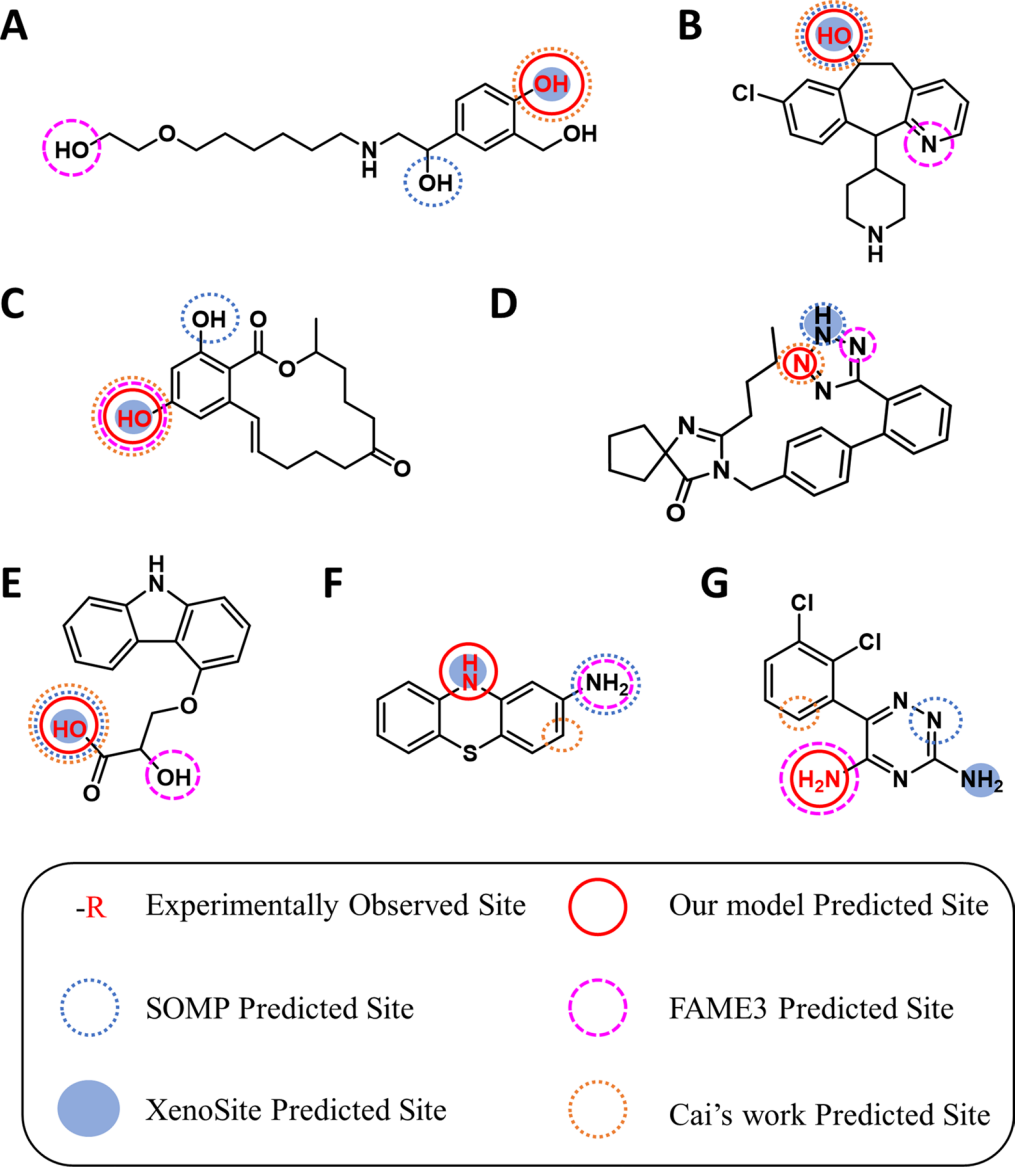
Some examples (e.g. compounds **A–G**) of top-1 prediction results of different models (our model, SOMP, FAME 3, XenoSite, Cai’s work) on the test set for the SOM prediction model

Figure 7 presented some examples of the top-1 possible SOM of UGT enzymes predicted by our model, SOMP, and FAME3. For example, Fig. 7D showed the predicted SOM by three models of Irbesartan [33], which is an angiotensin II receptor antagonist and is used to treat hypertension. Glucuronidation of Irbesartan is one of the major routes of elimination. Compared with the other two models, our model can more accurately predict the site where the glucuronidation reaction may occur, which is more useful to provide valuable information for structural optimization and improve the pharmacokinetic properties of drugs.

## Discussion

### Combination model for substrate/nonsubstrate prediction model

In this study, substrate prediction models for UGT enzymes were built by traditional ML methods and GNN methods. The traditional ML methods were based on molecular fingerprints and physicochemical descriptors, where we should calculate molecular fingerprints and physicochemical descriptors before model building. However, the GNN methods would extract the molecular features by the convolution layers, so we don’t need to calculate any descriptors by other programs. All the data were collected from literature, and we developed fast, simple and effective models to predict the reactivity of molecules and UGT enzymes.

We built single models including traditional models and GNN models, and we selected the top-10 models based on the performance of tenfold cross-validation. As shown in the results (Fig. 5 and Additional file 1: Table S10) of tenfold cross-validation can find that the GNN model and traditional ML models performed quite well. Although the data size was not big, the GNN model still had a good performance.

In order to improve the stability of the model, we selected the top-10 models based on MCC metrics of tenfold cross-validation to construct consensus models which can predict if a molecule is the substrate of UGT enzymes. We built 1023 consensus models which combined n models from the top 10 models. According to the result of MCC and AUC metrics in the test set shown in Table 2 and Table 3, the consensus model had improved than the single model. The traditional ML models were based on fingerprint and physicochemical descriptors, while the GNN models were based on molecular graphs. As shown in Table 3, we found that the molecular representation of the top-3 consensus models consisted of physicochemical descriptors, fingerprints, and molecular graphs. In order to further explore the justifiability of the consensus model, we tried to build consensus model only with traditional ML model or GNNs. Interestingly, consensus model which combined traditional ML models and GNNs can improve the performance than the best consensus model built only with traditional ML models or GNNs. It indicated that the combination of different molecular representation types contributed to the model performance because consensus models learned more information from the different single models. Thus, it can not only improve the performance of the model but also improve the robustness to build a consensus model. we chose the top-1 consensus model as the final substrate prediction model.

### The discussion of the SOM prediction model firstly built with GNN methods

After predicting if a molecule is the substrate of UGT enzymes, the model for the site of metabolism prediction was established by the WLN model to predict where the site of metabolism of the substrate is located. The WLN model has some advantages. Firstly, it uses graphs to represent molecular features without calculating complex descriptors by other programs. Secondly, this model only needs the information on the reaction metabolized by UGT enzymes. Thirdly, the WLN model is interpretable as it is consistent with the analysis of metabolites by chemists. Coley et al. designed a state-of-the-art neural model to be aligned with how domain experts (chemists) might analyze a problem, and the WLN model applied global attention mechanism to offer interpretability [16]. Last but not least, compared with the global metabolic model, the local model has higher accuracy and can provide more accurate help for medicinal chemists. Our model can still achieve better results with a small amount of data. As shown in Fig. 6C, with the growth of the amount of data, the top-1, top-2, and top-3 accuracy of the model gradually increased, so the performance of our model will also improve with the increase in data on UGTs metabolism in the future. As shown in Additional file 1: Fig. S4, we found that the molecules which were wrongly predicted at first-ranked site were large and complex. Most of the molecules had multiple potential sites which can be metabolized by UGTs (–O or –N), and the atom environment of SOMs which were wrongly predicted was more similar to the atom environment of true SOMs in the training set, so it’s difficult to predict the SOM for complex molecules which had multiple potential SOMs. Even so, these molecules could be correctly predicted at the second-ranked site.

We compared our model with SOMP, FAME3, XenoSite, and Cai’s work on the same test set, and we found our model had better performance than the others. Since the data of the XenoSite was extracted from the Accelrys Metabolite Database which is commercial, and our data was collected from literature manually, the data size of XenoSite is larger than ours. Nevertheless, both XenoSite and our model performed almost equally well on AUC and MCC metrics, and our top-1 accuracy still performed better than XenoSite. It can be explained that the WLN model has robust performance on a small data set.

Compared with our previous work presented by Cai et al. [9], this work has some improvements. Firstly, we built a prediction model for substrate/nonsubstrate before the prediction for SOM. Secondly, we collected more reaction data metabolized by UGT enzymes and our top-k accuracy results were better than before. Last but not least, we don’t need to calculate descriptors before building the model as the WLN model automatically extracts features through convolutional layers.

In some sense, we can only use the SOM model to predict. We found that most metabolic prediction models were constructed only on substrates, such as SOMP, XenoSite and our model. However, the sample distribution was calculated, and some nonsubstrates fell outside the application domain of SOM model. Thus, we built a model which can predict if a molecule is the substrate of the UGT enzyme and then predict the SOM of the substrate.

## Conclusions

In this study, we applied traditional ML and GNN methods to predict the UGT-mediated metabolism of drug-like molecules. For the substrate prediction, we selected the optimal models from both categories of methods according to evaluation indicators and then combined them to build consensus models to improve the predictive performance and stability. We applied the WLN method to predict the SOMs of UGT enzymes. It was the first time to use the GNN method in the prediction of SOMs, which didn’t need to calculate complex descriptors by other programs. Compared with other published models, our model can achieve a more accurate prediction performance on the same test dataset. As the number of data increases, the performance of deep learning models will improve. In a word, Meta-UGT would provide reasonable guidance for UGT enzyme-mediated metabolism, which is conducive to the optimization of pharmacokinetic properties of compounds in drug discovery.

## Supplementary Information

**Additional file 1: Figure S1**. The Mechanism of Glucuronidation. **Figure S2.** The MCC value of the Y-Randomization model and original method. **Figure S3. **A) Heatmap showing the the similarity among training set and test set for the substrate/nonsubstrate model; B) Heatmap showing the the similarity among training set and test set for the SOM model. **Figure S4.** The wrongly predicted molecules by the SOM model. **Table S1.** The definition of canonical atom feature for substrate prediction. **Table S2.** The definition of canonical bond feature for substrate prediction. **Table S3.** The definition of attentivefp atom feature for substrate prediction. **Table S4.** The definition of attentivefp bond feature for substrate prediction. **Table S5. **The definition of atom features for the WLN model (SOM prediction model). **Table S6.** The definition of bond features for the WLN model (SOM prediction model). **Table S7.** The tuned parameters of different traditional ML models for substrate prediction.** Table S8. **The best parameters of different traditional ML models for substrate prediction. **Table S9.** The parameters of GNN models for substrate prediction. **Table S10.** The performance in the training dataset of the substrate prediction model. **Table S11.** The performance of the 10-fold cross-validation of the substrate prediction model. **Table S12.** The results of the remaining 65 models in the test set of the substrate prediction model. **Table S13**. Configuration for the SOM model. **Table S14**. Statistical information for the three data sets of SOMs.

## Data Availability

All data used in this study are available at https://github.com/mengtinghuang/Meta-UGT. This GitHub repository also contains Jupyter notebooks and python scripts to reproduce the results and figures. The commercial software used to execute the study includes Pipeline Pilot, which was purchased by East China University of Science and Technology and licensed from BIOVIA (https://www.3ds.com/products-services/biovia/products/data-science/pipeline-pilot/). All the traditional ML methods were employed in the open-source ML toolkit scikit-learn (version 0.24.2) (https://scikit-learn.org/stable/), and all the GNN methods were trained by the Deep Graph Library (DGL) package (version 0.7.1) with CUDA 10.1 and the open-source model—DGL-LifeSci (version 0.2.8) (https://github.com/awslabs/dgl-lifesci/). All GNN models ran on the GPU version of the PyTorch framework (version 1.8.1).

## Abbreviations

UGTs: UDP-glucuronosyltransferases
ML: Machine learning
GNN: Graph neural network
SOM: Site of metabolism
Aliphatic hydroxyls: AlOH
Aromatic hydroxyls: ArOH
Carboxylic acids: COOH
Nitrogen containing sites: Nitrogen
WLN: Weisfeiler-Lehman Network
SMILES: Simplified Molecular Input Line System
RF: Random forest
SVM: Support vector machine
LR: Logistic regression
NN: Neural network
ET: Extremely randomized trees
GCN: Graph convolutional networks
GAT: Graph attention networks
MPNN: Message passing neural network
ACC: Accuracy
SE: Sensitivity
SP: Specificity
MCC: Matthews correlation coefficient
AUC: Area under the curve
TP: True positive
FP: False positive
TN: True negative
FN: False negative
PCA: Principal component analysis
