# Supplementary material for: In silico prediction of UGT-mediated metabolism in drug-like molecules via graph neural network
Source: J Cheminform. 2022 Jul 8;14:46. doi: 10.1186/s13321-022-00626-3 (PMC9270812; doi:10.1186/s13321-022-00626-3)
Supplement: Supplementary file 1 — Additional file 1: Figure S1. The Mechanism of Glucuronidation. Figure S2. The MCC value of the Y-Randomization model and original method. Figure S3. A) Heatmap showing the the similarity among training set and test set for the substrate/nonsubstrate model; B) Heatmap showing the the similarity among training set and test set for the SOM model. Figure S4. The wrongly predicted molecules by the SOM model. Table S1. The definition of canonical atom feature for substrate prediction. Table S2. The definition of canonical bond feature for substrate prediction. Table S3. The definition of attentivefp atom feature for substrate prediction. Table S4. The definition of attentivefp bond feature for substrate prediction. Table S5. The definition of atom features for the WLN model (SOM prediction model). Table S6. The definition of bond features for the WLN model (SOM prediction model). Table S7. The tuned parameters of different traditional ML models for substrate prediction. Table S8. The best parameters of different traditional ML models for substrate prediction. Table S9. The parameters of GNN models for substrate prediction. Table S10. The performance in the training dataset of the substrate prediction model. Table S11. The performance of the 10-fold cross-validation of the substrate prediction model. Table S12. The results of the remaining 65 models in the test set of the substrate prediction model. Table S13. Configuration for the SOM model. Table S14. Statistical information for the three data sets of SOMs. [file 13321_2022_626_MOESM1_ESM.docx]

***In Silico* Prediction of UGT-Mediated Metabolism in Drug-like Molecules via Graph Neural Network**

Mengting Huang, Chaofeng Lou, Zengrui Wu, Weihua Li, Philip W. Lee, Yun Tang*, Guixia Liu*

Shanghai Frontiers Science Center of Optogenetic Techniques for Cell Metabolism, School of Pharmacy, East China University of Science and Technology, Shanghai 200237, China

* To whom correspondence should be addressed.

Tel: +86-21-64250811

Fax: +86-21-64251033

Email: gxliu@ecust.edu.cn (G. Liu), ytang234@ecust.edu.cn (Y. Tang)

**Figure Legends**

**Figure S1**. The Mechanism of Glucuronidation.

**Figure S2.** The MCC value of the Y-Randomization model and original method.

**Figure S3.** A) Heatmap showing the the similarity among training set and test set for the substrate/nonsubstrate model; B) Heatmap showing the the similarity among training set and test set for the SOM model.

**Figure S4.** The wrongly predicted molecules by the SOM model.

**Table Legends**

**Table S1.** The definition of canonical atom feature for substrate prediction

**Table S2.** The definition of canonical bond feature for substrate prediction

**Table S3.** The definition of attentivefp atom feature for substrate prediction

**Table S4.** The definition of attentivefp bond feature for substrate prediction

**Table S5.** The definition of atom features for the WLN model (SOM prediction model)

**Table S6.** The definition of bond features for the WLN model (SOM prediction model)

**Table S7.** The tuned parameters of different traditional ML models for substrate prediction

**Table S8.** The best parameters of different traditional ML models for substrate prediction

**Table S9.** The parameters of GNN models for substrate prediction

**Table S10.** The performance in the training dataset of the substrate prediction model

**Table S11.** The performance of the 10-fold cross-validation of the substrate prediction model

**Table S12.** The results of the remaining 65 models in the test set of the substrate prediction model

**Table S13**. Configuration for the SOM model

**Table S14**. Statistical information for the three data sets of SOMs

**
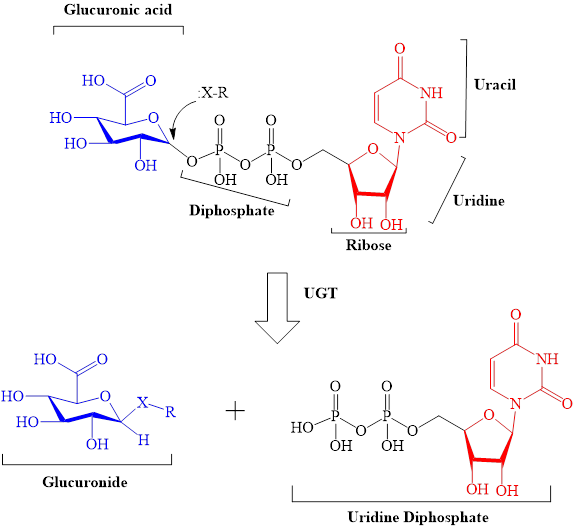
**

**Figure S1.** The Mechanism of Glucuronidation.


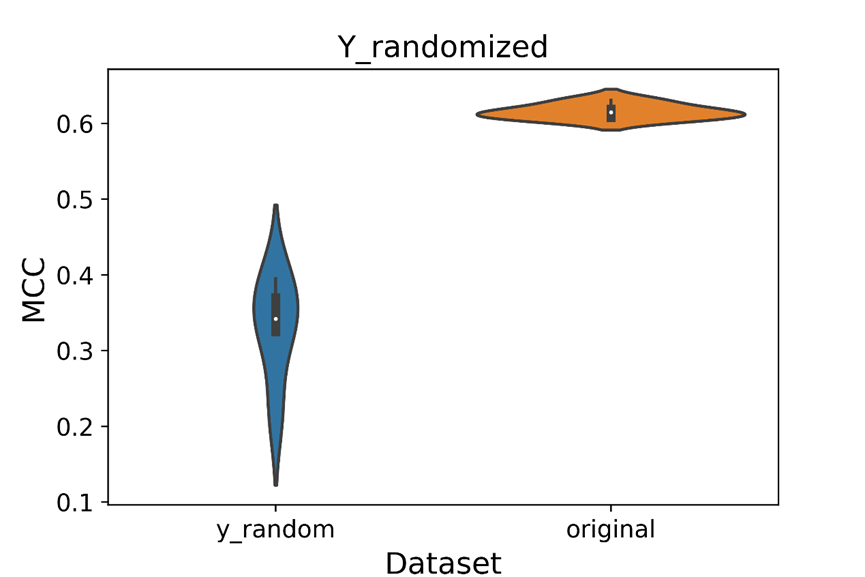


**Figure S2**. The MCC value of Y-Randomization model and original method.


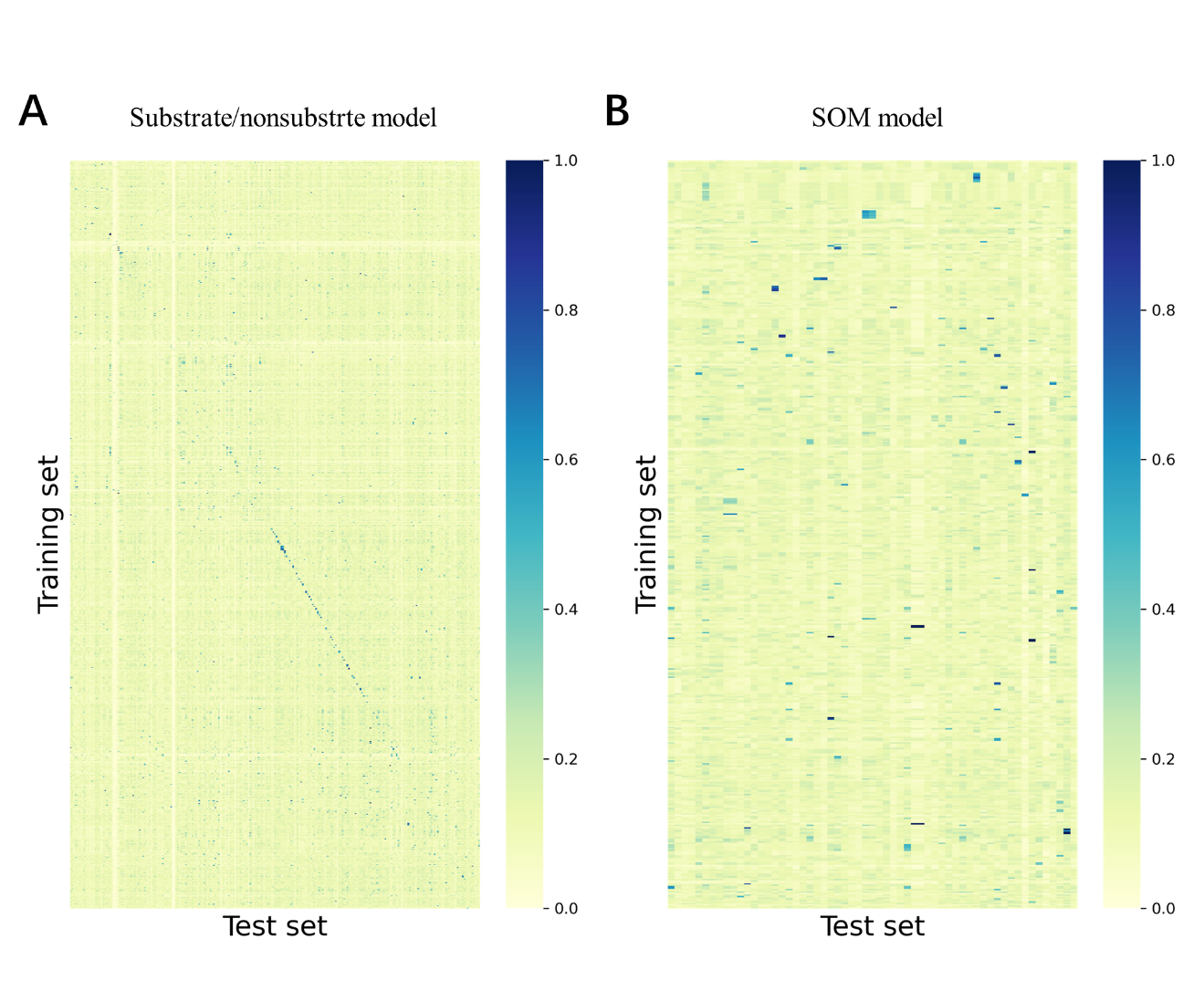


**Figure S3.** A) Heatmap showing the Tanimoto similarity among training set and test set for the substrate/nonsubstrate model; B) Heatmap showing the Tanimoto similarity among training set and test set for the SOM model.


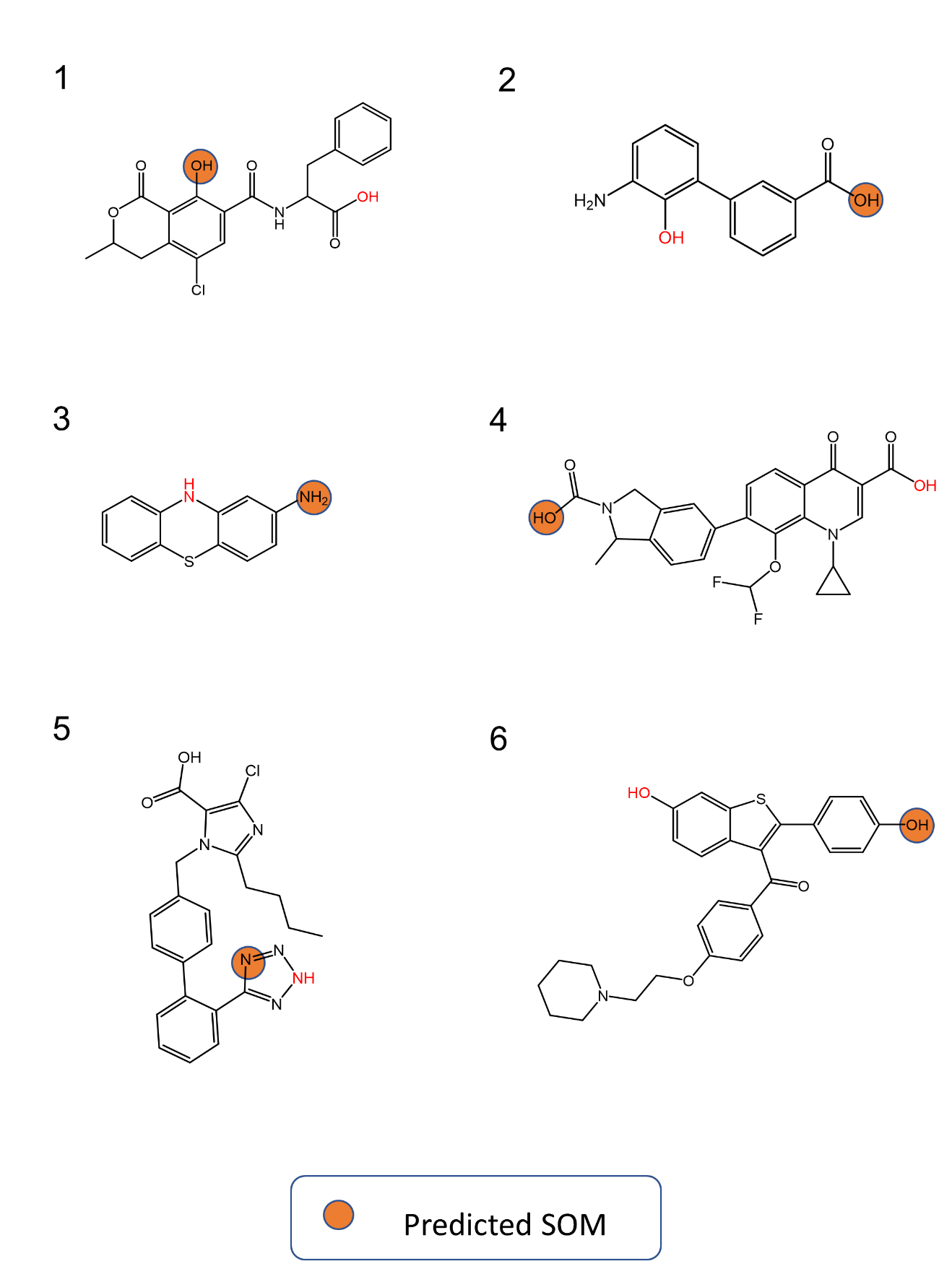


**Figure S4**. The wrongly predicted molecules by the SOM model.

**Table S1.** The definition of canonical atom feature for substrate prediction

| Atom feature | Description |
| --- | --- |
| Atom type | One hot vector specifying the type of this atom: ['C', 'N', 'O', 'S', 'F', 'Si', 'P', 'Cl', 'Br', 'Mg', 'Na', 'Ca', 'Fe', 'As', 'Al', 'I', 'B', 'V', 'K', 'Tl',  'Yb', 'Sb', 'Sn', 'Ag', 'Pd', 'Co', 'Se', 'Ti', 'Zn',  'H', 'Li', 'Ge', 'Cu', 'Au', 'Ni', 'Cd', 'In', 'Mn', 'Zr',  'Cr', 'Pt', 'Hg', 'Pb'] |
| Degree | 0-10 |
| Implicit valence | 0-6 |
| Hybridization | 'SP', 'SP2', 'SP3', 'SP3D', 'SP3D2' |
| Formal charge | The formal charge of the atom |
| In aromatic ring | Whether the atom is aromatic |
| the number of total Hs | 0 - 4 |

**Table S2.** The definition of canonical bond feature for substrate prediction

| Bond feature | Description |
| --- | --- |
| Bond type | One hot vector of Single, Double, Triple, Aromatic |
| Conjugated | whether the bond is conjugated |
| In rings | whether the bond is in a ring of any size |
| Stereo configuration | ‘Stereonone’, ‘Stereoany’, ‘Stereoz’, ‘Stereoe’,’Stereocis’, ‘Stereotrans’ |

**Table S3.** The definition of attentivefp atom feature for substrate prediction

| Atom feature | Description |
| --- | --- |
| Atom type | One hot vector specifying the type of this atom: ['B’, ‘C’, ‘N’, ‘O’, ‘F’, ‘Si’, ‘P’, ‘S’, ‘Cl’, ‘As’, ‘Se’, ‘Br’, ‘Te’, ‘I’, ‘At’, and ‘other’.] |
| Degree | 0-5 |
| Radical electrons | Number of radical electrons of the atom |
| Hybridization | 'SP', 'SP2', 'SP3', 'SP3D', 'SP3D2' and 'other' |
| Chiral center | Whether the atom is a chiral center |
| Formal charge | The formal charge of the atom |
| In aromatic ring | Whether the atom is aromatic |
| the number of total Hs | 0 - 4 |
| Chirality type | ‘R’, ‘S’ |

**Table S4.** The definition of attentivefp bond feature for substrate prediction

| Bond feature | Description |
| --- | --- |
| Bond type | One hot vector of Single, Double, Triple, Aromatic |
| Conjugated | whether the bond is conjugated |
| In rings | whether the bond is in a ring of any size |
| Stereo configuration | ‘Stereonone’, ‘Stereoany’, ‘Stereoz’, ‘Stereoe’,’Stereocis’, ‘Stereotrans’ |

**Table S5.** The definition of atom features for WLN model (SOM prediction model)

| Atom feature | Description |
| --- | --- |
| Atom type | One hot vector specifying the type of this atom: ['C', 'N', 'O', 'S', 'F', 'Si', 'P', 'Cl', 'Br', 'Mg', 'Na', 'Ca', 'Fe','As', 'Al', 'I', 'B', 'V', 'K', 'Tl', 'Yb', 'Sb', 'Sn', 'Ag', 'Pd', 'Co', 'Se', 'Ti', 'Zn', 'H', 'Li', 'Ge', 'Cu', 'Au', 'Ni', 'Cd', 'In', 'Mn', 'Zr', 'Cr', 'Pt', 'Hg', 'Pb', 'W', 'Ru', 'Nb', 'Re', 'Te', 'Rh', 'Tc', 'Ba', 'Bi', 'Hf', 'Mo', 'U', 'Sm', 'Os', 'Ir', 'Ce', 'Gd', 'Ga', 'Cs'] |
| Charge | Electrostatic charge of this atom: [-3, -2, -1, 0, 1, 2] |
| Degree | The degree of the atom: range (5) |
| Explicit valence | Explicit valence of an atom: range (1,6) |
| Implicit valence | Implicit valence of the atom: range (5) |
| In aromatic ring | whether the atom is aromatic |

**Table S6.** The definition of bond features for the WLN model (SOM prediction model)

| Bond feature | Description |
| --- | --- |
| Bond type | One hot vector of Single, Double, Triple, Aromatic |
| Conjugated | whether the bond is conjugated |
| In rings | whether the bond is in a ring of any size |

**Table S7.** The tuned parameters of different traditional ML models for substrate prediction

| Methods | Tuning parameters |
| --- | --- |
| RF | ’class_weight, 'criterion'', 'n_estimators', 'oob_score' |
| SVM | 'C', 'class_weight', 'gamma' |
| NN | 'activation', 'alpha', 'hidden_layer_sizes', 'learning_rate', 'max_iter', 'solver' |
| LR | 'C', 'solver' |
| ET | 'criterion', 'max_depth', 'n_estimators' |

**Table S8.** The best parameters of different traditional ML models for substrate prediction

| Methods | Tuning parameters |
| --- | --- |
| Descriptor_RF | ‘class_weight': 'balanced', 'criterion': 'gini', 'n_estimators': 80, 'oob_score': 'True' |
| Descriptor_SVM | ’C': 32.0, 'class_weight': 'balanced', 'gamma': 0.03125 |
| Descriptor_NN | 'activation': 'relu', 'alpha': 0.001, 'hidden_layer_sizes': (100,), 'learning_rate': 'constant', 'max_iter': 10000, 'solver': 'adam' |
| Descriptor_LR | 'C': 1.51, 'solver': 'lbfgs' |
| Descriptor_ET | 'criterion': 'entropy', 'max_depth': 20, 'n_estimators': 100 |
| MACCS_RF | 'class_weight': 'balanced', 'criterion': 'gini', 'n_estimators': 80. |
| MACCS_SVM | C': 32.0, 'class_weight': 'balanced', 'gamma': 0.03125, 'kernel': 'rbf'. |
| MACCS_NN | 'activation': 'relu', 'alpha': 0.001, 'hidden_layer_sizes': (100,), 'learning_rate': 'constant', 'max_iter': 10000, 'solver': 'adam'. |
| MACCS_LR | 'C': 1.51, 'solver': 'lbfgs'. |
| MACCS_ET | 'criterion': 'entropy', 'max_depth': 20, 'n_estimators': 100. |
| Morgan_RF_512 | class_weight': 'balanced_subsample', 'criterion': 'gini', 'n_estimators': 80 |
| Morgan_RF_1024 | class_weight': 'balanced', 'criterion': 'entropy', 'n_estimators': 100 |
| Morgan_RF_2048 | class_weight': 'balanced_subsample', 'criterion': 'gini', 'n_estimators': 80 |
| AtomPairs_RF_512 | class_weight': 'balanced_subsample', 'criterion': 'gini', 'n_estimators': 120 |
| AtomPairs_RF_1024 | class_weight': 'balanced_subsample', 'criterion': 'entropy', 'n_estimators': 80 |
| AtomPairs_RF_2048 | class_weight': 'balanced_subsample', 'criterion': 'entropy', 'n_estimators': 90 |
| TopoTorsion_RF_512 | class_weight': 'balanced', 'criterion': 'entropy', 'n_estimators': 30 |
| TopoTorsion_RF_1024 | class_weight': 'balanced_subsample', 'criterion': 'gini', 'n_estimators': 50 |
| TopoTorsion_RF_2048 | class_weight': 'balanced', 'criterion': 'entropy', 'n_estimators': 110 |
| Morgan_SVM_512 | C': 8.0, 'class_weight': 'balanced', 'gamma': 0.03125, 'kernel': 'rbf' |
| Morgan_SVM_1024 | C': 2.0, 'class_weight': 'balanced', 'gamma': 0.0078125, 'kernel': 'rbf' |
| Morgan_SVM_2048 | C': 32.0, 'class_weight': 'balanced', 'gamma': 0.03125, 'kernel': 'rbf' |
| AtomPairs_SVM_512 | C': 8.0, 'class_weight': 'balanced', 'gamma': 0.03125, 'kernel': 'rbf' |
| AtomPairs_SVM_1024 | C': 2.0, 'class_weight': 'balanced', 'gamma': 0.0078125, 'kernel': 'rbf' |
| AtomPairs_SVM_2048 | C': 32.0, 'class_weight': 'balanced', 'gamma': 0.0078125, 'kernel': 'rbf' |
| TopoTorsion_SVM_512 | C': 8.0, 'class_weight': 'balanced', 'gamma': 0.03125, 'kernel': 'rbf' |
| TopoTorsion_SVM_1024 | C': 8.0, 'class_weight': 'balanced', 'gamma': 0.03125, 'kernel': 'rbf' |
| TopoTorsion_SVM_2048 | C': 8.0, 'class_weight': 'balanced', 'gamma': 0.0078125, 'kernel': 'rbf' |
| Morgan_NN_512 | activation': 'relu', 'alpha': 0.1, 'hidden_layer_sizes': (500,), 'learning_rate': 'constant', 'max_iter': 10000, 'solver': 'adam' |
| Morgan_NN_1024 | activation': 'relu', 'alpha': 0.1, 'hidden_layer_sizes': (500,), 'learning_rate': 'constant', 'max_iter': 10000, 'solver': 'adam' |
| Morgan_NN_2048 | activation': 'relu', 'alpha': 0.1, 'hidden_layer_sizes': (300,), 'learning_rate': 'constant', 'max_iter': 10000, 'solver': 'adam' |
| AtomPairs_NN_512 | activation': 'relu', 'alpha': 0.01, 'hidden_layer_sizes': (300,), 'learning_rate': 'constant', 'max_iter': 10000, 'solver': 'adam' |
| AtomPairs_NN_1024 | activation': 'relu', 'alpha': 0.01, 'hidden_layer_sizes': (500,), 'learning_rate': 'constant', 'max_iter': 10000, 'solver': 'adam' |
| AtomPairs_NN_2048 | activation': 'relu', 'alpha': 1e-05, 'hidden_layer_sizes': (500,), 'learning_rate': 'constant', 'max_iter': 10000, 'solver': 'adam' |
| TopoTorsion_NN_512 | activation': 'relu', 'alpha': 0.1, 'hidden_layer_sizes': (500,), 'learning_rate': 'constant', 'max_iter': 10000, 'solver': 'adam' |
| TopoTorsion_NN_1024 | activation': 'relu', 'alpha': 1e-06, 'hidden_layer_sizes': (200,), 'learning_rate': 'constant', 'max_iter': 10000, 'solver': 'adam' |
| TopoTorsion_NN_2048 | activation': 'relu', 'alpha': 0.01, 'hidden_layer_sizes': (500,), 'learning_rate': 'constant', 'max_iter': 10000, 'solver': 'adam' |
| Morgan_LR_512 | C': 0.01, 'solver': 'lbfgs' |
| Morgan_LR_1024 | C': 0.51, 'solver': 'liblinear' |
| Morgan_LR_2048 | C': 0.51, 'solver': 'liblinear' |
| AtomPairs_LR_512 | C': 0.01, 'solver': 'lbfgs' |
| AtomPairs_LR_1024 | C': 0.51, 'solver': 'sag' |
| AtomPairs_LR_2048 | C': 0.01, 'solver': 'liblinear' |
| TopoTorsion_LR_512 | C': 0.51, 'solver': 'lbfgs' |
| TopoTorsion_LR_1024 | C': 1.01, 'solver': 'sag' |
| TopoTorsion_LR_2048 | C': 0.51, 'solver': 'liblinear' |
| Morgan_ET_512 | criterion': 'gini', 'max_depth': 25, 'n_estimators': 60 |
| Morgan_ET_1024 | criterion': 'gini', 'max_depth': 15, 'n_estimators': 80 |
| Morgan_ET_2048 | criterion': 'gini', 'max_depth': 25, 'n_estimators': 70 |
| AtomPairs_ET_512 | criterion': 'entropy', 'max_depth': 20, 'n_estimators': 30 |
| AtomPairs_ET_1024 | criterion': 'gini', 'max_depth': 25, 'n_estimators': 90 |
| AtomPairs_ET_2048 | criterion': 'entropy', 'max_depth': 15, 'n_estimators': 60 |
| TopoTorsion_ET_512 | criterion': 'gini', 'max_depth': 20, 'n_estimators': 100 |
| TopoTorsion_ET_1024 | criterion': 'gini', 'max_depth': 30, 'n_estimators': 30 |
| TopoTorsion_ET_2048 | criterion': 'entropy', 'max_depth': 15, 'n_estimators': 90 |
| RDKFingerprint_RF_512 | class_weight': 'balanced_subsample', 'criterion': 'gini', 'n_estimators': 80 |
| RDKFingerprint_RF_1024 | class_weight': 'balanced', 'criterion': 'gini', 'n_estimators': 120 |
| RDKFingerprint_RF_2048 | class_weight': 'balanced', 'criterion': 'entropy', 'n_estimators': 100 |
| RDKFingerprint_NN_512 | activation': 'relu', 'alpha': 1e-05, 'hidden_layer_sizes': (200,), 'learning_rate': 'constant', 'max_iter': 10000, 'solver': 'adam' |
| RDKFingerprint_NN_1024 | activation': 'relu', 'alpha': 0.0001, 'hidden_layer_sizes': (100,), 'learning_rate': 'constant', 'max_iter': 10000, 'solver': 'adam' |
| RDKFingerprint_NN_2048 | activation': 'relu', 'alpha': 0.0001, 'hidden_layer_sizes': (200,), 'learning_rate': 'constant', 'max_iter': 10000, 'solver': 'adam' |
| RDKFingerprint_LR_512 | C': 1.51, 'solver': 'lbfgs' |
| RDKFingerprint_LR_1024 | C': 1.01, 'solver': 'lbfgs' |
| RDKFingerprint_LR_2048 | C': 0.51, 'solver': 'newton-cg' |
| RDKFingerprint_ET_512 | criterion': 'gini', 'max_depth': 30, 'n_estimators': 40 |
| RDKFingerprint_ET_1024 | criterion': 'entropy', 'max_depth': 25, 'n_estimators': 60 |
| RDKFingerprint_ET_2048 | criterion': 'gini', 'max_depth': 10, 'n_estimators': 100 |
| RDKFingerprint_SVM_512 | C': 32.0, 'class_weight': 'balanced', 'gamma': 0.03125, 'kernel': 'rbf' |
| RDKFingerprint_SVM_1024 | C': 32.0, 'class_weight': 'balanced', 'gamma': 0.001953125, 'kernel': 'rbf' |
| RDKFingerprint_SVM_2048 | C': 128.0, 'class_weight': 'balanced', 'gamma': 3.0517578125e-05, 'kernel': 'rbf' |

Methods name includes the type of features and the type of machine learning methods. MACCS fingerprint was fixed 166 bits. Other four types fingerprint had different sizes including 512, 1024 and 2048 bits.

**Table S9.** The parameters of GNN models for substrate prediction

| Methods | Feature type | Parameters | |
| --- | --- | --- | --- |
| GCN | canonical | | "lr": 0.02, "batch_size": 128, "dropout": 0.05, "gnn_hidden_feats": 256, "predictor_hidden_feats": 128, "num_gnn_layers": 2, "residual": true, "batchnorm": false, "in_node_feats": 74 |
| GAT | canonical | | "lr": 0.0003, "batch_size": 128, "dropout": 0.05, "gnn_hidden_feats": 64, "num_heads": 8, "alpha": 0.06, "predictor_hidden_feats": 128, "num_gnn_layers": 5, "in_node_feats": 74 |
| Weave | canonical | | "lr": 0.0003, "batch_size": 128, "num_gnn_layers": 5, "gnn_hidden_feats": 50, "graph_feats": 128, "gaussian_expand": true, "in_node_feats": 74, "in_edge_feats": 13 |
| MPNN | canonical | | "lr": 0.0003, "batch_size": 128, "node_out_feats": 64, "edge_hidden_feats": 128, "num_step_message_passing": 6, "num_step_set2set": 6, "num_layer_set2set": 3, "in_node_feats": 74, "in_edge_feats": 13 |
| AttentiveFP | canonical | | "lr": 0.0003, "batch_size": 128, "num_layers": 2, "num_timesteps": 2, "graph_feat_size": 200, "dropout": 0, "in_node_feats": 74, "in_edge_feats": 13 |
| GCN | attentivefp | | "lr": 0.02, "batch_size": 128, "dropout": 0.05, "gnn_hidden_feats": 256, "predictor_hidden_feats": 128, "num_gnn_layers": 2, "residual": true, "batchnorm": false, "in_node_feats": 39 |
| GAT | attentivefp | | "lr": 0.0003, "batch_size": 128, "dropout":0.05, "gnn_hidden_feats": 64, "num_heads": 8, "alpha": 0.06, "predictor_hidden_feats": 128, "num_gnn_layers": 5, "residual": true, "in_node_feats": 39 |
| Weave | attentivefp | | "lr": 0.0003, "batch_size": 128, "num_gnn_layers": 5, "gnn_hidden_feats": 50, "graph_feats": 128, "gaussian_expand": true, "in_node_feats": 39, "in_edge_feats": 11 |
| MPNN | attentivefp | | "lr": 0.0003, "batch_size": 128, "node_out_feats": 64, "edge_hidden_feats": 128, "num_step_message_passing": 6, "num_step_set2set": 6, "num_layer_set2set": 3, "in_node_feats": 39, "in_edge_feats": 11 |
| AttentiveFP | attentivefp | | "lr": 0.0003, "batch_size": 128, "num_layers": 2, "num_timesteps": 2, "graph_feat_size": 200, "dropout": 0, "in_node_feats": 39, "in_edge_feats": 11 |

**Table S10.** The performance in the training dataset of the substrate prediction model

| Method | ACC | SE | AUC | MCC | SP |
| --- | --- | --- | --- | --- | --- |
| MACCS_RF | 0.983 | 0.984 | 0.995 | 0.966 | 0.981 |
| MACCS_SVM | 0.986 | 0.992 | 0.991 | 0.972 | 0.979 |
| MACCS_NN | 0.981 | 0.990 | 0.991 | 0.962 | 0.971 |
| MACCS_LR | 0.859 | 0.868 | 0.930 | 0.717 | 0.849 |
| MACCS_ET | 0.982 | 0.984 | 0.998 | 0.964 | 0.979 |
| Descriptor_SVM | 0.887 | 0.898 | 0.954 | 0.774 | 0.876 |
| Descriptor_NN | 0.976 | 0.971 | 0.994 | 0.952 | 0.981 |
| Descriptor_LR | 0.856 | 0.875 | 0.926 | 0.712 | 0.836 |
| Descriptor_RF | 0.981 | 0.975 | 0.993 | 0.962 | 0.988 |
| Descriptor_ET | 0.983 | 0.977 | 0.996 | 0.966 | 0.990 |
| RDKFingerprint_RF_512 | 0.968 | 0.971 | 0.992 | 0.936 | 0.965 |
| RDKFingerprint_RF_1024 | 0.976 | 0.977 | 0.994 | 0.952 | 0.975 |
| RDKFingerprint_RF_2048 | 0.974 | 0.977 | 0.991 | 0.948 | 0.971 |
| Morgan_RF_512 | 0.980 | 0.981 | 0.997 | 0.960 | 0.979 |
| Morgan_RF_1024 | 0.985 | 0.984 | 0.996 | 0.970 | 0.986 |
| Morgan_RF_2048 | 0.985 | 0.984 | 0.996 | 0.970 | 0.986 |
| AtomPairs_RF_512 | 0.982 | 0.984 | 0.998 | 0.964 | 0.979 |
| AtomPairs_RF_1024 | 0.979 | 0.981 | 0.997 | 0.958 | 0.977 |
| AtomPairs_RF_2048 | 0.980 | 0.984 | 0.998 | 0.960 | 0.975 |
| TopoTorsion_RF_512 | 0.979 | 0.979 | 0.992 | 0.958 | 0.979 |
| TopoTorsion_RF_1024 | 0.979 | 0.977 | 0.994 | 0.958 | 0.981 |
| TopoTorsion_RF_2048 | 0.976 | 0.971 | 0.995 | 0.952 | 0.981 |
| RDKFingerprint_NN_512 | 0.957 | 0.979 | 0.982 | 0.915 | 0.934 |
| RDKFingerprint_NN_1024 | 0.966 | 0.959 | 0.988 | 0.932 | 0.973 |
| RDKFingerprint_NN_2048 | 0.972 | 0.979 | 0.991 | 0.944 | 0.965 |
| Morgan_NN_512 | 0.981 | 0.981 | 0.993 | 0.962 | 0.981 |
| Morgan_NN_1024 | 0.981 | 0.990 | 0.993 | 0.962 | 0.971 |
| Morgan_NN_2048 | 0.985 | 0.981 | 0.993 | 0.970 | 0.990 |
| AtomPairs_NN_512 | 0.973 | 0.979 | 0.993 | 0.946 | 0.967 |
| AtomPairs_NN_1024 | 0.979 | 0.975 | 0.993 | 0.958 | 0.983 |
| AtomPairs_NN_2048 | 0.983 | 0.984 | 0.993 | 0.966 | 0.981 |
| TopoTorsion_NN_512 | 0.979 | 0.979 | 0.993 | 0.958 | 0.979 |
| TopoTorsion_NN_1024 | 0.974 | 0.965 | 0.990 | 0.948 | 0.983 |
| TopoTorsion_NN_2048 | 0.975 | 0.965 | 0.993 | 0.950 | 0.986 |
| RDKFingerprint_LR_512 | 0.928 | 0.924 | 0.967 | 0.856 | 0.932 |
| RDKFingerprint_LR_1024 | 0.969 | 0.961 | 0.985 | 0.938 | 0.977 |
| RDKFingerprint_LR_2048 | 0.976 | 0.977 | 0.991 | 0.952 | 0.975 |
| Morgan_LR_512 | 0.831 | 0.831 | 0.909 | 0.661 | 0.831 |
| Morgan_LR_1024 | 0.965 | 0.971 | 0.989 | 0.930 | 0.959 |
| Morgan_LR_2048 | 0.976 | 0.979 | 0.992 | 0.952 | 0.973 |
| AtomPairs_LR_512 | 0.794 | 0.806 | 0.881 | 0.587 | 0.781 |
| AtomPairs_LR_1024 | 0.961 | 0.963 | 0.987 | 0.922 | 0.959 |
| AtomPairs_LR_2048 | 0.854 | 0.860 | 0.933 | 0.707 | 0.847 |
| TopoTorsion_LR_512 | 0.860 | 0.858 | 0.926 | 0.720 | 0.862 |
| TopoTorsion_LR_1024 | 0.933 | 0.938 | 0.973 | 0.866 | 0.928 |
| TopoTorsion_LR_2048 | 0.945 | 0.946 | 0.980 | 0.890 | 0.944 |
| RDKFingerprint_ET_512 | 0.963 | 0.981 | 0.993 | 0.926 | 0.944 |
| RDKFingerprint_ET_1024 | 0.975 | 0.975 | 0.996 | 0.950 | 0.975 |
| RDKFingerprint_ET_2048 | 0.977 | 0.979 | 0.995 | 0.954 | 0.975 |
| Morgan_ET_512 | 0.982 | 0.983 | 0.997 | 0.964 | 0.981 |
| Morgan_ET_1024 | 0.975 | 0.961 | 0.994 | 0.950 | 0.990 |
| Morgan_ET_2048 | 0.981 | 0.981 | 0.995 | 0.962 | 0.981 |
| AtomPairs_ET_512 | 0.979 | 0.983 | 0.998 | 0.958 | 0.975 |
| AtomPairs_ET_1024 | 0.981 | 0.983 | 0.998 | 0.962 | 0.979 |
| AtomPairs_ET_2048 | 0.978 | 0.977 | 0.997 | 0.956 | 0.979 |
| TopoTorsion_ET_512 | 0.980 | 0.979 | 0.996 | 0.960 | 0.981 |
| TopoTorsion_ET_1024 | 0.980 | 0.975 | 0.996 | 0.960 | 0.986 |
| TopoTorsion_ET_2048 | 0.950 | 0.918 | 0.991 | 0.902 | 0.983 |
| RDKFingerprint_SVM_512 | 0.964 | 0.967 | 0.995 | 0.928 | 0.961 |
| RDKFingerprint_SVM_1024 | 0.967 | 0.965 | 0.993 | 0.934 | 0.969 |
| RDKFingerprint_SVM_2048 | 0.935 | 0.942 | 0.983 | 0.870 | 0.928 |
| Morgan_SVM_512 | 0.984 | 0.984 | 0.995 | 0.968 | 0.983 |
| Morgan_SVM_1024 | 0.910 | 0.897 | 0.974 | 0.820 | 0.924 |
| Morgan_SVM_2048 | 0.984 | 0.986 | 0.994 | 0.968 | 0.981 |
| AtomPairs_SVM_512 | 0.979 | 0.979 | 0.998 | 0.958 | 0.979 |
| AtomPairs_SVM_1024 | 0.970 | 0.965 | 0.995 | 0.940 | 0.975 |
| AtomPairs_SVM_2048 | 0.982 | 0.979 | 0.992 | 0.964 | 0.986 |
| TopoTorsion_SVM_512 | 0.975 | 0.969 | 0.993 | 0.950 | 0.981 |
| TopoTorsion_SVM_1024 | 0.971 | 0.961 | 0.992 | 0.942 | 0.981 |
| TopoTorsion_SVM_2048 | 0.953 | 0.944 | 0.988 | 0.906 | 0.963 |

**Table S11.** The performance of 10-fold cross-validation of the substrate prediction model

| Method | ACC | STD | SE | STD | AUC | STD | MCC | STD | SP | STD |
| --- | --- | --- | --- | --- | --- | --- | --- | --- | --- | --- |
| Descriptor_ET | 0.837 | 0.017 | 0.865 | 0.048 | 0.905 | 0.025 | 0.678 | 0.034 | 0.807 | 0.06 |
| Descriptor_RF | 0.835 | 0.028 | 0.858 | 0.064 | 0.907 | 0.025 | 0.673 | 0.058 | 0.811 | 0.05 |
| MACCS_ET | 0.832 | 0.026 | 0.85 | 0.048 | 0.888 | 0.031 | 0.665 | 0.052 | 0.812 | 0.048 |
| Descriptor_SVM | 0.827 | 0.035 | 0.842 | 0.073 | 0.895 | 0.033 | 0.657 | 0.069 | 0.811 | 0.056 |
| MACCS_RF | 0.827 | 0.03 | 0.846 | 0.046 | 0.892 | 0.025 | 0.655 | 0.059 | 0.806 | 0.046 |
| Descriptor_LR | 0.824 | 0.033 | 0.85 | 0.069 | 0.883 | 0.034 | 0.651 | 0.067 | 0.797 | 0.058 |
| MACCS_NN | 0.825 | 0.03 | 0.841 | 0.046 | 0.886 | 0.023 | 0.651 | 0.059 | 0.808 | 0.057 |
| AttentiveFP_attentivefp | 0.821 | 0.019 | 0.837 | 0.042 | 0.898 | 0.016 | 0.644 | 0.038 | 0.802 | 0.036 |
| MACCS_SVM | 0.82 | 0.033 | 0.845 | 0.05 | 0.884 | 0.024 | 0.642 | 0.067 | 0.793 | 0.055 |
| Morgan_ET_1024 | 0.817 | 0.026 | 0.844 | 0.042 | 0.885 | 0.019 | 0.636 | 0.051 | 0.787 | 0.068 |
| AttentiveFP_canonical | 0.816 | 0.028 | 0.818 | 0.068 | 0.898 | 0.020 | 0.635 | 0.054 | 0.814 | 0.055 |
| Morgan_RF_2048 | 0.815 | 0.034 | 0.840 | 0.060 | 0.883 | 0.024 | 0.632 | 0.067 | 0.787 | 0.048 |
| Morgan_LR_2048 | 0.815 | 0.033 | 0.833 | 0.044 | 0.890 | 0.016 | 0.631 | 0.067 | 0.795 | 0.060 |
| Descriptor_NN | 0.813 | 0.031 | 0.822 | 0.069 | 0.884 | 0.020 | 0.630 | 0.064 | 0.803 | 0.070 |
| AtomPairs_ET_2048 | 0.814 | 0.033 | 0.842 | 0.043 | 0.878 | 0.025 | 0.629 | 0.064 | 0.783 | 0.057 |
| GCN_attentivefp | 0.810 | 0.023 | 0.795 | 0.088 | 0.896 | 0.010 | 0.628 | 0.039 | 0.826 | 0.079 |
| Morgan_ET_2048 | 0.812 | 0.031 | 0.829 | 0.048 | 0.880 | 0.019 | 0.625 | 0.062 | 0.793 | 0.053 |
| Morgan_NN_2048 | 0.811 | 0.041 | 0.804 | 0.052 | 0.882 | 0.021 | 0.624 | 0.083 | 0.818 | 0.064 |
| Morgan_RF_1024 | 0.811 | 0.036 | 0.835 | 0.056 | 0.883 | 0.023 | 0.623 | 0.072 | 0.785 | 0.045 |
| Morgan_SVM_2048 | 0.811 | 0.034 | 0.833 | 0.045 | 0.887 | 0.018 | 0.623 | 0.067 | 0.787 | 0.064 |
| AtomPairs_RF_2048 | 0.807 | 0.032 | 0.858 | 0.055 | 0.874 | 0.029 | 0.616 | 0.066 | 0.752 | 0.035 |
| GCN_canonical | 0.804 | 0.016 | 0.771 | 0.067 | 0.897 | 0.009 | 0.614 | 0.028 | 0.839 | 0.046 |
| AtomPairs_ET_1024 | 0.805 | 0.034 | 0.835 | 0.055 | 0.870 | 0.025 | 0.612 | 0.066 | 0.772 | 0.061 |
| AtomPairs_SVM_2048 | 0.804 | 0.027 | 0.810 | 0.046 | 0.873 | 0.024 | 0.608 | 0.053 | 0.798 | 0.037 |
| Morgan_SVM_512 | 0.803 | 0.038 | 0.823 | 0.062 | 0.878 | 0.021 | 0.607 | 0.074 | 0.781 | 0.048 |
| Morgan_ET_512 | 0.803 | 0.026 | 0.827 | 0.039 | 0.873 | 0.019 | 0.606 | 0.051 | 0.777 | 0.039 |
| MPNN_canonical | 0.798 | 0.022 | 0.863 | 0.056 | 0.883 | 0.015 | 0.602 | 0.040 | 0.728 | 0.079 |
| Morgan_RF_512 | 0.800 | 0.029 | 0.839 | 0.050 | 0.878 | 0.022 | 0.602 | 0.058 | 0.758 | 0.052 |
| MPNN_attentivefp | 0.799 | 0.021 | 0.813 | 0.049 | 0.873 | 0.017 | 0.600 | 0.041 | 0.785 | 0.050 |
| MACCS_LR | 0.799 | 0.029 | 0.825 | 0.043 | 0.865 | 0.028 | 0.599 | 0.057 | 0.771 | 0.052 |
| AtomPairs_SVM_1024 | 0.799 | 0.039 | 0.813 | 0.052 | 0.869 | 0.027 | 0.598 | 0.077 | 0.783 | 0.058 |
| GAT_attentivefp | 0.797 | 0.015 | 0.835 | 0.061 | 0.887 | 0.020 | 0.598 | 0.033 | 0.757 | 0.049 |
| AtomPairs_RF_1024 | 0.797 | 0.022 | 0.850 | 0.050 | 0.870 | 0.024 | 0.597 | 0.045 | 0.739 | 0.050 |
| Morgan_SVM_1024 | 0.797 | 0.038 | 0.792 | 0.062 | 0.879 | 0.019 | 0.595 | 0.075 | 0.802 | 0.043 |
| AtomPairs_NN_1024 | 0.792 | 0.031 | 0.796 | 0.042 | 0.846 | 0.045 | 0.584 | 0.061 | 0.787 | 0.048 |
| AtomPairs_NN_2048 | 0.791 | 0.032 | 0.815 | 0.046 | 0.853 | 0.031 | 0.582 | 0.065 | 0.764 | 0.053 |
| AtomPairs_ET_512 | 0.789 | 0.025 | 0.811 | 0.064 | 0.853 | 0.027 | 0.580 | 0.051 | 0.764 | 0.054 |
| AtomPairs_SVM_512 | 0.788 | 0.031 | 0.819 | 0.049 | 0.859 | 0.033 | 0.576 | 0.061 | 0.754 | 0.044 |
| AtomPairs_RF_512 | 0.785 | 0.032 | 0.833 | 0.061 | 0.842 | 0.035 | 0.572 | 0.065 | 0.733 | 0.045 |
| GAT_canonical | 0.783 | 0.030 | 0.806 | 0.098 | 0.867 | 0.022 | 0.570 | 0.053 | 0.757 | 0.058 |
| Morgan_LR_1024 | 0.784 | 0.024 | 0.786 | 0.046 | 0.870 | 0.017 | 0.569 | 0.048 | 0.781 | 0.045 |
| Morgan_NN_1024 | 0.782 | 0.039 | 0.794 | 0.079 | 0.866 | 0.019 | 0.566 | 0.077 | 0.769 | 0.043 |
| Morgan_LR_512 | 0.775 | 0.033 | 0.788 | 0.055 | 0.851 | 0.028 | 0.551 | 0.067 | 0.760 | 0.041 |
| AtomPairs_LR_2048 | 0.775 | 0.036 | 0.802 | 0.048 | 0.846 | 0.039 | 0.550 | 0.072 | 0.746 | 0.063 |
| Morgan_NN_512 | 0.775 | 0.032 | 0.808 | 0.038 | 0.860 | 0.022 | 0.550 | 0.064 | 0.740 | 0.057 |
| TopoTorsion_ET_2048 | 0.763 | 0.038 | 0.751 | 0.064 | 0.826 | 0.042 | 0.529 | 0.078 | 0.775 | 0.066 |
| TopoTorsion_SVM_2048 | 0.764 | 0.029 | 0.763 | 0.034 | 0.843 | 0.034 | 0.529 | 0.059 | 0.765 | 0.054 |
| AtomPairs_LR_1024 | 0.763 | 0.023 | 0.784 | 0.037 | 0.824 | 0.045 | 0.526 | 0.045 | 0.739 | 0.052 |
| Weave_canonical | 0.739 | 0.075 | 0.690 | 0.216 | 0.882 | 0.022 | 0.522 | 0.106 | 0.792 | 0.237 |
| TopoTorsion_ET_1024 | 0.760 | 0.037 | 0.777 | 0.053 | 0.825 | 0.048 | 0.521 | 0.074 | 0.742 | 0.066 |
| AtomPairs_NN_512 | 0.759 | 0.038 | 0.778 | 0.066 | 0.820 | 0.042 | 0.519 | 0.076 | 0.738 | 0.057 |
| TopoTorsion_RF_2048 | 0.757 | 0.047 | 0.759 | 0.070 | 0.834 | 0.047 | 0.515 | 0.093 | 0.754 | 0.060 |
| TopoTorsion_NN_2048 | 0.756 | 0.039 | 0.765 | 0.047 | 0.828 | 0.045 | 0.513 | 0.080 | 0.746 | 0.072 |
| TopoTorsion_ET_512 | 0.753 | 0.051 | 0.777 | 0.047 | 0.821 | 0.049 | 0.507 | 0.102 | 0.727 | 0.093 |
| TopoTorsion_SVM_1024 | 0.752 | 0.032 | 0.757 | 0.048 | 0.824 | 0.039 | 0.505 | 0.064 | 0.746 | 0.065 |
| RDKFingerprint_RF_1024 | 0.749 | 0.030 | 0.796 | 0.060 | 0.808 | 0.027 | 0.500 | 0.061 | 0.698 | 0.059 |
| TopoTorsion_LR_2048 | 0.750 | 0.028 | 0.765 | 0.026 | 0.819 | 0.033 | 0.499 | 0.058 | 0.733 | 0.055 |
| RDKFingerprint_NN_2048 | 0.747 | 0.030 | 0.759 | 0.102 | 0.807 | 0.026 | 0.499 | 0.054 | 0.733 | 0.075 |
| TopoTorsion_NN_1024 | 0.749 | 0.037 | 0.753 | 0.046 | 0.810 | 0.036 | 0.499 | 0.075 | 0.744 | 0.068 |
| RDKFingerprint_ET_2048 | 0.748 | 0.039 | 0.773 | 0.081 | 0.813 | 0.032 | 0.498 | 0.079 | 0.721 | 0.055 |
| TopoTorsion_RF_1024 | 0.747 | 0.039 | 0.765 | 0.060 | 0.821 | 0.044 | 0.496 | 0.078 | 0.727 | 0.069 |
| RDKFingerprint_RF_2048 | 0.746 | 0.024 | 0.788 | 0.051 | 0.817 | 0.019 | 0.493 | 0.050 | 0.700 | 0.052 |
| RDKFingerprint_NN_1024 | 0.744 | 0.039 | 0.773 | 0.055 | 0.800 | 0.039 | 0.490 | 0.079 | 0.713 | 0.087 |
| RDKFingerprint_SVM_1024 | 0.744 | 0.040 | 0.753 | 0.068 | 0.812 | 0.031 | 0.490 | 0.079 | 0.733 | 0.080 |
| RDKFingerprint_ET_1024 | 0.743 | 0.034 | 0.752 | 0.065 | 0.808 | 0.028 | 0.487 | 0.066 | 0.733 | 0.043 |
| RDKFingerprint_SVM_2048 | 0.741 | 0.043 | 0.773 | 0.073 | 0.799 | 0.045 | 0.485 | 0.087 | 0.707 | 0.078 |
| Weave_attentivefp | 0.721 | 0.091 | 0.802 | 0.239 | 0.878 | 0.013 | 0.483 | 0.140 | 0.634 | 0.270 |
| AtomPairs_LR_512 | 0.740 | 0.021 | 0.765 | 0.046 | 0.809 | 0.046 | 0.480 | 0.042 | 0.713 | 0.052 |
| TopoTorsion_SVM_512 | 0.738 | 0.044 | 0.753 | 0.045 | 0.816 | 0.045 | 0.476 | 0.089 | 0.721 | 0.079 |
| TopoTorsion_NN_512 | 0.735 | 0.057 | 0.724 | 0.080 | 0.801 | 0.040 | 0.472 | 0.114 | 0.746 | 0.077 |
| TopoTorsion_RF_512 | 0.735 | 0.042 | 0.747 | 0.049 | 0.792 | 0.043 | 0.470 | 0.084 | 0.721 | 0.055 |
| RDKFingerprint_LR_2048 | 0.733 | 0.040 | 0.748 | 0.070 | 0.794 | 0.038 | 0.468 | 0.080 | 0.717 | 0.071 |
| RDKFingerprint_ET_512 | 0.729 | 0.032 | 0.759 | 0.071 | 0.792 | 0.027 | 0.459 | 0.064 | 0.696 | 0.048 |
| RDKFingerprint_LR_1024 | 0.724 | 0.034 | 0.722 | 0.058 | 0.770 | 0.029 | 0.451 | 0.069 | 0.725 | 0.085 |
| TopoTorsion_LR_1024 | 0.724 | 0.032 | 0.730 | 0.050 | 0.786 | 0.028 | 0.448 | 0.064 | 0.717 | 0.052 |
| RDKFingerprint_RF_512 | 0.723 | 0.046 | 0.751 | 0.069 | 0.793 | 0.037 | 0.446 | 0.091 | 0.692 | 0.035 |
| TopoTorsion_LR_512 | 0.708 | 0.041 | 0.714 | 0.059 | 0.757 | 0.036 | 0.416 | 0.083 | 0.700 | 0.065 |
| RDKFingerprint_SVM_512 | 0.707 | 0.033 | 0.771 | 0.062 | 0.771 | 0.030 | 0.416 | 0.067 | 0.638 | 0.065 |
| RDKFingerprint_NN_512 | 0.698 | 0.054 | 0.724 | 0.097 | 0.759 | 0.038 | 0.398 | 0.109 | 0.669 | 0.065 |
| RDKFingerprint_LR_512 | 0.659 | 0.048 | 0.655 | 0.064 | 0.695 | 0.042 | 0.320 | 0.097 | 0.664 | 0.077 |

**Table S12.** The results of the remaining 65 models in the test set of the substrate prediction model

| Model | Acc | SP | SE | AUC | MCC |
| --- | --- | --- | --- | --- | --- |
| AttentiveFP_canonical | 0.775 | 0.867 | 0.876 | 0.557 | 0.678 |
| Morgan_RF_2048 | 0.831 | 0.793 | 0.867 | 0.896 | 0.663 |
| Morgan_LR_2048 | 0.791 | 0.828 | 0.885 | 0.582 | 0.752 |
| AtomPairs_ET_2048 | 0.815 | 0.752 | 0.875 | 0.870 | 0.633 |
| Descriptor_NN | 0.783 | 0.820 | 0.868 | 0.566 | 0.744 |
| GCN_attentivefp | 0.803 | 0.736 | 0.867 | 0.881 | 0.609 |
| Morgan_ET_2048 | 0.807 | 0.760 | 0.852 | 0.883 | 0.615 |
| Morgan_NN_2048 | 0.787 | 0.820 | 0.884 | 0.574 | 0.752 |
| Morgan_RF_1024 | 0.807 | 0.727 | 0.883 | 0.889 | 0.619 |
| Morgan_SVM_2048 | 0.819 | 0.777 | 0.859 | 0.894 | 0.639 |
| AtomPairs_RF_2048 | 0.819 | 0.744 | 0.891 | 0.864 | 0.643 |
| GCN_canonical | 0.779 | 0.875 | 0.865 | 0.565 | 0.678 |
| AtomPairs_ET_1024 | 0.787 | 0.867 | 0.875 | 0.579 | 0.702 |
| AtomPairs_SVM_2048 | 0.799 | 0.852 | 0.874 | 0.600 | 0.744 |
| Morgan_SVM_512 | 0.835 | 0.769 | 0.898 | 0.884 | 0.674 |
| Morgan_ET_512 | 0.835 | 0.744 | 0.922 | 0.890 | 0.679 |
| MPNN_canonical | 0.791 | 0.852 | 0.862 | 0.584 | 0.727 |
| Morgan_RF_512 | 0.831 | 0.777 | 0.883 | 0.883 | 0.665 |
| MPNN_attentivefp | 0.783 | 0.867 | 0.836 | 0.572 | 0.694 |
| MACCS_LR | 0.803 | 0.828 | 0.859 | 0.606 | 0.777 |
| AtomPairs_SVM_1024 | 0.795 | 0.859 | 0.865 | 0.593 | 0.727 |
| GAT_attentivefp | 0.811 | 0.752 | 0.867 | 0.870 | 0.625 |
| AtomPairs_RF_1024 | 0.803 | 0.702 | 0.898 | 0.874 | 0.615 |
| Morgan_SVM_1024 | 0.791 | 0.828 | 0.875 | 0.582 | 0.752 |
| AtomPairs_NN_1024 | 0.779 | 0.836 | 0.859 | 0.560 | 0.719 |
| AtomPairs_NN_2048 | 0.779 | 0.797 | 0.860 | 0.558 | 0.760 |
| AtomPairs_ET_512 | 0.747 | 0.836 | 0.821 | 0.498 | 0.653 |
| AtomPairs_SVM_512 | 0.771 | 0.859 | 0.848 | 0.548 | 0.678 |
| AtomPairs_RF_512 | 0.767 | 0.891 | 0.838 | 0.547 | 0.636 |
| GAT_canonical | 0.795 | 0.828 | 0.849 | 0.590 | 0.760 |
| Morgan_LR_1024 | 0.775 | 0.828 | 0.863 | 0.551 | 0.719 |
| Morgan_NN_1024 | 0.751 | 0.859 | 0.861 | 0.510 | 0.636 |
| Morgan_LR_512 | 0.779 | 0.820 | 0.857 | 0.558 | 0.736 |
| AtomPairs_LR_2048 | 0.779 | 0.797 | 0.824 | 0.558 | 0.760 |
| Morgan_NN_512 | 0.791 | 0.852 | 0.876 | 0.584 | 0.727 |
| TopoTorsion_ET_2048 | 0.751 | 0.766 | 0.828 | 0.501 | 0.736 |
| TopoTorsion_SVM_2048 | 0.767 | 0.805 | 0.830 | 0.534 | 0.727 |
| AtomPairs_LR_1024 | 0.759 | 0.828 | 0.819 | 0.520 | 0.686 |
| Weave_canonical | 0.819 | 0.793 | 0.844 | 0.865 | 0.638 |
| TopoTorsion_ET_1024 | 0.767 | 0.852 | 0.826 | 0.539 | 0.678 |
| AtomPairs_NN_512 | 0.763 | 0.898 | 0.836 | 0.542 | 0.620 |
| TopoTorsion_RF_2048 | 0.791 | 0.852 | 0.847 | 0.584 | 0.727 |
| TopoTorsion_NN_2048 | 0.755 | 0.789 | 0.818 | 0.510 | 0.719 |
| TopoTorsion_ET_512 | 0.755 | 0.859 | 0.832 | 0.518 | 0.645 |
| TopoTorsion_SVM_1024 | 0.763 | 0.820 | 0.842 | 0.527 | 0.702 |
| RDKFingerprint_RF_1024 | 0.771 | 0.828 | 0.847 | 0.543 | 0.711 |
| TopoTorsion_LR_2048 | 0.751 | 0.797 | 0.807 | 0.502 | 0.702 |
| RDKFingerprint_NN_2048 | 0.791 | 0.875 | 0.867 | 0.588 | 0.702 |
| RDKFingerprint_ET_2048 | 0.803 | 0.711 | 0.891 | 0.860 | 0.613 |
| AtomPairs_LR_512 | 0.711 | 0.661 | 0.758 | 0.774 | 0.421 |
| TopoTorsion_RF_1024 | 0.763 | 0.844 | 0.833 | 0.530 | 0.678 |
| RDKFingerprint_RF_2048 | 0.795 | 0.922 | 0.863 | 0.607 | 0.661 |
| RDKFingerprint_NN_1024 | 0.759 | 0.734 | 0.832 | 0.520 | 0.785 |
| RDKFingerprint_SVM_1024 | 0.763 | 0.820 | 0.838 | 0.527 | 0.702 |
| RDKFingerprint_ET_1024 | 0.743 | 0.852 | 0.832 | 0.494 | 0.628 |
| RDKFingerprint_SVM_2048 | 0.763 | 0.828 | 0.844 | 0.528 | 0.694 |
| Weave_attentivefp | 0.787 | 0.922 | 0.863 | 0.592 | 0.645 |
| TopoTorsion_RF_512 | 0.747 | 0.813 | 0.810 | 0.495 | 0.678 |
| RDKFingerprint_LR_2048 | 0.775 | 0.820 | 0.843 | 0.551 | 0.727 |
| RDKFingerprint_ET_512 | 0.755 | 0.820 | 0.832 | 0.512 | 0.686 |
| RDKFingerprint_LR_1024 | 0.759 | 0.813 | 0.808 | 0.519 | 0.702 |
| TopoTorsion_SVM_512 | 0.727 | 0.645 | 0.805 | 0.821 | 0.456 |
| TopoTorsion_NN_512 | 0.719 | 0.612 | 0.820 | 0.795 | 0.443 |
| RDKFingerprint_LR_2048 | 0.775 | 0.727 | 0.820 | 0.843 | 0.551 |
| TopoTorsion_LR_1024 | 0.727 | 0.678 | 0.773 | 0.798 | 0.454 |
| RDKFingerprint_RF_512 | 0.719 | 0.661 | 0.773 | 0.813 | 0.438 |
| TopoTorsion_LR_512 | 0.691 | 0.653 | 0.727 | 0.769 | 0.381 |
| RDKFingerprint_SVM_512 | 0.699 | 0.579 | 0.813 | 0.775 | 0.403 |
| RDKFingerprint_NN_512 | 0.743 | 0.669 | 0.813 | 0.815 | 0.488 |
| RDKFingerprint_LR_512 | 0.679 | 0.636 | 0.719 | 0.752 | 0.356 |

The methods name of GNN model means GNN methods type and features type.

**Table S13**. Configuration for SOM model

| Parameters | Value |
| --- | --- |
| Batch size | 20 |
| Hidden size | 300 |
| Max norm | 5.0 |
| Node in feats | 82 |
| Edge in feats | 6 |
| Node pair in feats | 10 |
| Learning rate | 0.001 |
| Decay every | 50 |
| Learning rate decay factor | 0.9 |

**Table S14**. Statistical information for the three data sets of SOMs

| Type | Training set | Validation set | Test set |
| --- | --- | --- | --- |
| AlOH | 124 | 17 | 11 |
| ArOH | 207 | 23 | 20 |
| COOH | 102 | 11 | 17 |
| Nitrogen | 102 | 9 | 12 |
